# Multipotent Systems: Combining Planning, Self-Organization, and Reconfiguration in Modular Robot Ensembles

**DOI:** 10.3390/s19010017

**Published:** 2018-12-20

**Authors:** Oliver Kosak, Constantin Wanninger, Alwin Hoffmann, Hella Ponsar, Wolfgang Reif

**Affiliations:** Institute for Software & Systems Engineering, University of Augsburg, Augsburg 86159, Germany; wanninger@isse.de (C.W.); hoffmann@isse.de (A.H.); ponsar@isse.de (H.P.); reif@isse.de (W.R.)

**Keywords:** reconfiguration, multiagent systems, multipotent systems, self-awareness, self-organization, planning, semantic hardware, semantic plug and play, swarm, mobile robotics

## Abstract

Mobile multirobot systems play an increasing role in many disciplines. Their capabilities can be used, e.g., to transport workpieces in industrial applications or to support operational forces in search and rescue scenarios, among many others. Depending on the respective application, the hardware design and accompanying software of mobile robots are of various forms, especially for integrating different sensors and actuators. Concerning this design, robots of one system compared to each other can be classified to exclusively be either homogeneous or heterogeneous, both resulting in different system properties. While homogeneously configured systems are known to be robust against failures through redundancy but are highly specialized for specific use cases, heterogeneously designed systems can be used for a broad range of applications but suffer from their specialization, i.e., they can only hardly compensate for the failure of one specialist. Up to now, there has been no known approach aiming to unify the benefits of both these types of system. In this paper, we present our approach to filling this gap by introducing a reference architecture for mobile robots that defines the interplay of all necessary technologies for achieving this goal. We introduce the class of robot systems implementing this architecture as *multipotent systems* that bring together the benefits of both system classes, enabling homogeneously designed robots to become heterogeneous specialists at runtime. When many of these robots work together, we call the structure of this cooperation an *ensemble*. To achieve multipotent ensembles, we also integrate reconfigurable and self-descriptive hardware (i.e., sensors and actuators) in this architecture, which can be freely combined to change the capabilities of robots at runtime. Because typically a high degree of autonomy in such systems is a prerequisite for their practical usage, we also present the integration of necessary mechanisms and algorithms for achieving the systems’ multipotency. We already achieved the first results with robots implementing our approach of multipotent systems in real-world experiments as well as in a simulation environment, which we present in this paper.

## 1. Introduction

Human capabilities are limited. This concerns acting as well as sensing capabilities, each expressed to a certain and varying degree for each individual. On the one hand, this is obvious for acting in a physical environment, e.g., when trying to move heavy objects, which can quickly become too exhausting. On the other hand, while it is commonly known that the first categorization concerning human sensing capabilities was developed by Greek philosophers Aristotle [1] and Democritus [2] (the traditional senses of *touch*, *hearing*, *sight*, *smell*, and *taste*), it is outdated in modern physiology. Nevertheless, while more sensing capabilities are ascribed to humans (e.g., thermoception and internal senses, like the vestibular sense for body balance and acceleration) [3], there are still senses found by science that are not available to humans (e.g., ultrasonic echolocation of bats [4], electroreceptor sensor pores of sharks [5]). Additionally, the available sensing capabilities of most humans are not as precisely pronounced as they are in other species (e.g., dogs’ sense for smell is much finer-grained [6]). This insight combined with creative ideas on how to beneficially use non-inherent senses serves as a permanent motivation for humankind to develop new technologies. In these days, to compensate for the many deficiencies concerning our acting and sensing capabilities, intelligent systems like robots become representatives, providing capabilities which are desirable in certain situations. Applications range from involving robots dedicated to heavyweight object manipulation tasks in automobile production [7] to robots providing support in daily life [8] and to highly flexible mobile robots, like unmanned aerial vehicles (UAV) providing camera-enabled bird-view perspectives [9] (optionally in infrared [10]). Recently, those robots have also been enabled to cooperatively work together for achieving even better results on more complex tasks, e.g., for search and rescue [11,12,13,14], distributed surveillance of critical infrastructure [15,16,17], or environmental research [18,19,20]. Technologies enabling this cooperation can be found in many research disciplines. Those range from complex planning and coordination approaches to determine useful action sequences [21], over so-called swarm intelligence technologies [22,23] exploiting local interactions between robots (emergence [24]) to enable robust and scalable executions, to pioneering inventions concerning the design and programming of microsized modular hardware used [25,26,27].

**Figure 1:**
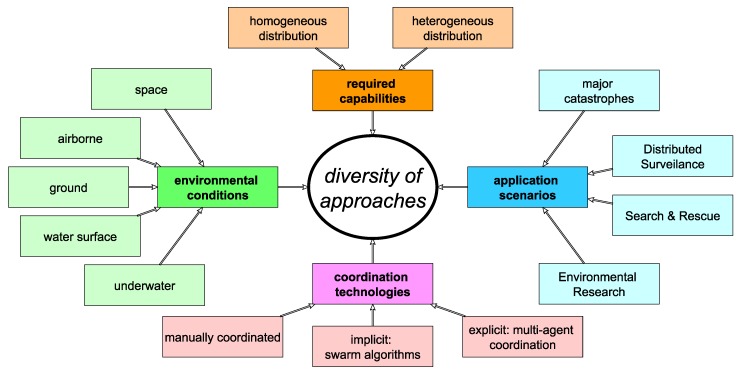
An excerpt of reasons causing the diversity of approaches found in literature concerning research done on technologies dedicated to autonomous (multi-) robot systems.

From an observing perspective, many inventions currently often address similar problems (or tasks) in different granularity, being adapted to diverse environmental conditions (e.g., space [28], airborne [12,14], on-ground [13], water surface level [18], or underwater [19]), application scenarios (cf. above, e.g., environmental research [18,19,20], search and rescue [13,14,28], distributed surveillance [15,16,29,30], or major catastrophes [12,31,32]), the systems’ configurations (homogeneous [18,22,33] or heterogeneous [13,14,19,23,31] distribution of capabilities among robots), and coordination technologies (implicitly done with, e.g., swarm algorithms [18,19,23], explicitly done with, e.g., multiagent technologies [13,14,31] or manual coordination [20]). All these aspects lead to a high diversity of approaches (cf. Figure 1).

For each new use case, the wheel is reinvented over and over again, and new robots with new capabilities for new applications have to be designed, built, and programmed with individualized software (cf. current approaches [13,14,15,18]). This strategy has multiple drawbacks:

- *(D1) Resource and engineering overhead*: The amount of needed robots grows proportionally with the number of different tasks and use cases.
- *(D2) Tradeoff between robustness or versatility*: On the one hand, within heterogeneous systems, the heterogeneity in robots leads to reduced robustness against failures, i.e., if a specialized robot breaks down, it has to be replaced by an equivalently configured one. Homogeneous systems, on the other hand, are specialists in solving one dedicated task and cannot be used within others.
- *(D3) Planning and allocation complexity*: Creating plans that define the procedure of execution for tasks is NP-complete [34], while allocating those tasks to robots is NP-hard, worsened through “larger team sizes and greater heterogeneity of robots and tasks” [35].
- *(D4) Proprietary software solutions*: In many projects, the software, i.e., the realized capabilities of the robot system, is developed for exactly the given use cases and the used set of hardware, e.g., [19,26,36]. Changes in the requirements or the used hardware usually entail high software engineering efforts. Systems with reconfigurable capabilities, however, are at the moment very restricted in their expandability [25] and development [37] or they act exclusively in a simulated environment [38].
- *(D5) Specialized hardware platform*: Hardware platforms are highly dependent on the scenarios (e.g., [22,26,39]) and cannot be used for other purposes without serious engineering effort.

With the approach presented in this paper, we show a way to overcome these issues by separating capabilities from robots in multirobot systems by introducing reconfigurable, modular robots, homogeneous at design time, heterogeneous as needed at runtime. In the scope of our approach, a robot is a conglomerate of multiple hardware modules (sensors and actuators) attached to a platform that is able to autonomously make use of that attached hardware modules for deriving capabilities and accomplishing tasks by executing these capabilities. Many of those robots working together are called an *ensemble*. When we refer to the term (physical) reconfiguration, we use the following interpretation:

A *(physical) reconfiguration* is a modification, applied to the existing hardware configuration of one (or multiple) robots (i.e., the set of attached actuators and sensors) which can result in a changed set of capabilities for that robot(s) due to the modified configuration(s).

We use the terms homogeneity and heterogeneity to define the similarity of these hardware configurations (i.e., robots) to each other. In that terminology, homogeneous robots consist of the exact same hardware configuration, i.e., provide the same capabilities, while heterogeneous robots consist of different hardware configurations, i.e., may provide different capabilities. We name this new hybrid form of system class where of robots’ capabilities can be reconfigured on a physical level *multipotent systems*. This is an analogy from biological research where multipotent progenitor cells are known for their ability to adapt their functionality when brought into new environments [40]. In our approach, we aim at merging current homogeneous and heterogeneous systems and inheriting the benefits of both system classes while omitting their drawbacks. On the one hand, with their runtime heterogeneity, robots in multipotent systems can be configured very individually to reproduce the versatility of heterogeneous systems [41]. On the other hand, with their design time homogeneity, robots in multipotent systems easily can compensate for each other in case of failures, as can be done in homogeneous systems. Our approach therefore aims at developing a reference system architecture for ensembles consisting of multipotent robots to handle the broad class of ScORe missions [31], involving search (S), continuously observe (cO), and react (Re) tasks. This broad system class subsumes other types of applications, such as search and rescue [11], environmental research [18,19,39], distributed surveillance of critical infrastructure [15,16,17], or dealing with major catastrophic incidents [12]. Our focus therein is on aerial robots and mobile ground vehicles. To achieve our goals, we combine classical planning [42] with the paradigm of *self-organization* and *self-adaptation* [43] based on *self-descriptive*, *reconfigurable hardware*, and *robots*. We use the terms self-adaptation (SA) and self-organization (SO) following the definition of Reference [43]:

*Self-adaptive* systems work in a top–down manner. They evaluate their own global behavior and change it when the evaluation indicates that they are not accomplishing what they were intended to do, or when better functionality or performance is possible. Such systems typically operate with an explicit internal representation of themselves and their global goals.

*Self-organizing* systems work bottom–up. They are composed of a large number of components that interact according to simple and local rules. The global behavior of the system emerges from these local interactions, and it is difficult to deduce the properties of the global system by studying only the local properties of its parts. Such systems do not use internal representations of global properties or goals; they are often inspired by biological or sociological phenomena.

By combining these two approaches (top–down and bottom–up), we achieve a high autonomy and robustness in execution but still retain sufficient control of the system during planning.

The contribution of this particular work is to (1) reveal all general problem positions occurring when establishing multipotent systems and (2) elaborate and illustrate the interplay of all necessary technologies needed for enabling multipotent systems. Therefore, we put into context technologies we already developed, those that we are currently developing, and those that we plan to develop within our next steps.

In the following paragraphs, we first depict our case study and elaborate its challenges in Section 2. We further present how we counter the drawbacks mentioned above of current approaches for (multi-) robot systems in Section 3, present our concepts and ideas already found in Section 4, and present the proof of concepts and preliminary results we have achieved up to now in Section 5. In Section 6, we investigate related work and finally give a conclusion on our findings as well as an outlook on future research in Section 7.

## 2. Case Study and Its Challenges

**Figure 2:**
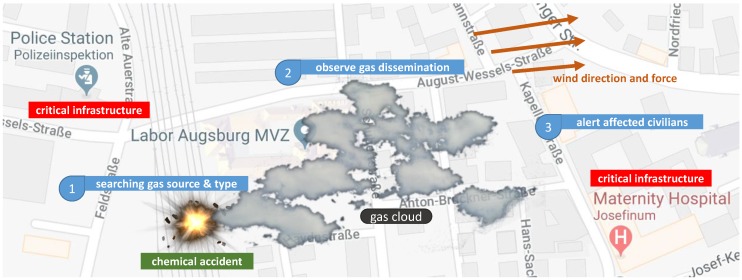
Gas accident on a railway in Augsburg city. The gas clouds’ spreading range is highly influenced by the wind direction and force. Dependent on the spreading range, only the right critical infrastructure (Maternity Hospital) is affected by the toxic gas cloud. The three steps illustrate the typical sequential procedure for firefighters to handle gas accidents.

Throughout our project and within this paper, we evaluate our findings with a chemical accident case study (cf. Figure 2). Unfortunately, such accidents happen quite often as current incidents at BASF in Germany (2016) [44], at Arkema in Texas (2017) [45], and Bayernoil in Germany (2018) [46] exemplarily demonstrate. Our case study stems from the domain of major catastrophic events, which typically require ScORe missions to be solved. In chemical accidents, firefighters often face the threat of toxic gases. First of all, the situation has to be clarified by searching (S) the relevant parameter (i.e., the gas with the highest risk potential as well as its source). After the identification of that parameter (e.g., a particular gas), its dissemination has to be continuously observed (cO) to enable estimations on its harmfulness. The observation can result in the need for appropriate reactions (Re), e.g., the evacuation of threatened inhabited areas. Currently, firefighters must make observations manually and take actions with limited, incomplete, or even wrong information [12]. For example, emergency forces do measurements of gas concentrations mainly at the location where the accident most likely happened and few additional other points close to the ground, due to limited available staff or difficult terrain. Therefore, firefighters need to go into endangered areas, possibly exposing themselves to hazardous gases. In addition, it is difficult to get a complete overview of the affected area only based on these few local observations. Despite these unfavorable circumstances, firefighters must rely on these measurements and identify gas types and concentrations, as well as roughly estimate the dimensions of the gas cloud. Using those rough estimations influenced by too few ground measurements, different expansion patterns of gases or possibly old weather data, the operational forces have to make far-reaching decisions on whether to evacuate nearby areas, e.g., residential houses, retirement homes, or even hospitals. To evacuate a particular area, a typical method for firefighters is to traverse inhabited streets, making loudspeaker announcements to the population. Staff availability permitting, they also ring doorbells, particularly in large buildings. Still, it is not easy to reach everyone in rural areas or urban recreation areas. Currently, an appropriate technique for getting an overview in such situations is also missing, e.g., for detecting people that are out for walks. A robot ensemble could substantially improve the way such accidents can be handled, as shown by previous work (cf., [12]), which already considered, e.g., gas cloud tracking using a swarm of Micro-UAVs. As shown in the example above, ScORe missions typically consist of multiple tasks (ScORe tasks) to be executed sequentially or in parallel. Moreover, there are dependencies between tasks and different requirements for the robot ensemble to successfully fulfill all tasks. For the first task S of the chemical gas accident example, the aerial robots need to be equipped with multigas sensors to identify the relevant gas, whereas for the next task cO, they need to be equipped with a more precise sensor for the detected gas. For a further Re task, e.g., some robots need to be equipped with cameras, loudspeakers, or signals to warn people. In addition to different task requirements, the ensemble is faced with uncertainties when dealing with ScORe missions. Examples for uncertainties are defects of robots at runtime, lack of clarity regarding the initial (environmental) setting the ensemble has to work in, and its development during runtime. These uncertainties make it hard to calculate a complete plan (including task scheduling and allocation) for each robot in advance. Complex ScORe missions call for adaptation at runtime. Our vision of an ensemble including self-aware and reconfigurable robots would go a step further and allow the ensemble to optimize its composition during such a mission to be able to deal, e.g., with subsequent tasks.

## 3. Objectives

The objective of our work is to overcome the five main drawbacks *(D1–D5)* of current approaches and literature introduced in Section 1, at least for the ScORe system class. Therefore, our approach follows the idea of combining classic planning approaches with self-organization mechanisms to enable the robot ensemble to adapt to unforeseen changes in the ensemble as well as in the environment and to react appropriately, without time-intensive replanning phases, and as autonomously as possible throughout all tasks of a ScORe mission. To equip a robot ensemble with the necessary degree of freedom, we propose to enhance existing hardware components with semantic knowledge and unified communication interfaces, to make robots self-aware through self-descriptive and reconfigurable hardware modules. This self-awareness allows the ensemble to reason from its current situation and optimize its state by initiating reconfigurations on the hardware and the software level at runtime. The goal of our approach, consequently, is to enable the handling of:

$$\begin{array}{c} \text{ScORe missions with self-aware robot ensembles through self-organization and planning},\\ \text{based on self-descriptive and reconfigurable hardware},\\ \text{enabling multipotent systems}. \end{array}$$

What ScORe missions have in common is that not all aspects of the environment are known in advance. In addition, it is assumed that the system should react to reoccurring situations found in the observed scenario and adapt its behavior accordingly. This behavior needs the system to be as autonomous as possible. We achieved this autonomy through self-organizing as we define it in Section 1. This type of autonomy can be exploited to execute previously generated plans as robustly as possible. The need for self-descriptive and reconfigurable hardware and the possibility of reasoning from it originates from the fact that we assume to have different ensembles and robot configurations during the course of one ScORe mission while we do not know the complete set of capabilities that will be needed to fulfill all tasks in advance.

**Figure 3:**
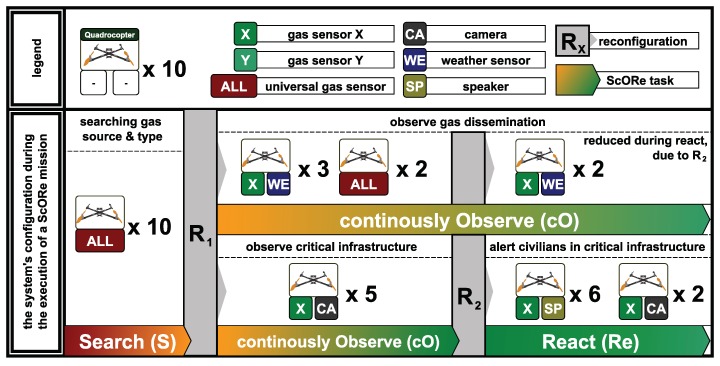
Simplified example of how a reconfigurable robot ensemble can execute a ScORe mission, e.g., a gas accident. The top part of the figure shows the available components (robots as well as additional sensors and actuators) and serves as a legend. The bottom part shows the changes in the system’s configuration (i.e., component compositions) in the course of the mission (dependencies between tasks are ordered from left to right, including parallel execution). $R_{1}$ and $R_{2}$ indicate reconfiguration phases of the ensemble [47].

Figure 3 shows how we envision an ensemble of reconfigurable robots to execute such a ScORe mission efficiently. We assume that in general, firefighters will have only a restricted set of hardware available due to the costs and the resulting maintenance efforts. The reconfigurability of hardware components will allow the ensemble to achieve maximum efficiency given a limited number of such expensive components, by recombining those with varying cheaper components, like sensors or speakers. In the example shown in Figure 3, the ensemble consists of ten quadrocopters and a variety of additional components, like sensors, cameras, and loudspeakers. Note that with the techniques embedded in our approach, we aim at a much higher number of components (e.g., 100 quadrocopters, at least in simulation) that we plan to employ in order to form highly efficient ensembles for large-scale ScORe missions. The initial task of the ScORe mission from Figure 3 is to determine the type of the predominant gas and search (S) for the source of toxic gases. For this purpose, all quadrocopters are best equipped with universal gas sensors to get a quick overview of the situation. Due to the size and weight of these sensors, each quadrocopter is fully loaded with this type of sensor. As soon as the predominant gas and its source is known, the next task in the ScORe mission requires a continuous observation (cO) of the gas cloud to be accomplished. In this case, the task is twofold (we refer to the scenario presented in Section 2): On the one hand, the dissemination of the toxic gas is to be observed, and on the other hand, critical infrastructures in the vicinity (e.g., a hospital) need to be particularly monitored. The reconfiguration phase ($R_{1}$) takes place to adapt the ensemble to the following requirements. The gas dissemination can be observed by focusing mainly on the most predominant gas (type ’X’ in the figure), for which smaller and lighter sensors are available. Measurements of the current local weather conditions (temperature, humidity, and wind) with additional lightweight sensors can help firefighters and the system in predicting the future expansion of the gas cloud. However, it is still essential to have a universal gas sensor at hand to be able to detect unforeseen additional gases. To monitor critical infrastructures, at least a sensor for the known predominant gas is necessary. Additionally, cameras can help firefighters to get an impression of the situation at the respective sites, e.g., regarding the density and movement of persons. In case the gas cloud expands to populated areas, evacuations have to be started—the react (Re) task of the ScORe mission automatically begins. In parallel to further observing the dissemination of gases (but with less quality), the ensemble autonomously begins to support the evacuation of affected areas by reconfiguring some quadrocopters ($R_{2}$) to carry loudspeakers in addition to their gas sensors. The speakers help to inform people about the evacuation quickly, in particular along the outside of high-rise buildings that firefighters otherwise would have to walk through. Cameras are still important to provide an aerial overview of the situation.

To enable robot ensembles to act autonomously this way in ScORe missions, and therefore tackle all drawbacks *(D1–D5)* from Section 1, our approach will deliver the following contributions *(C1–C5)*:

*(C1) Resource and engineering overhead:* For *(D1)*, we exploit the ability to reconfigure the ensemble’s robots regarding their capabilities. In comparison to the related literature [19,23,36,48], we do not need to provide appropriately configured robots for each of these tasks but enable the system to adapt to changing requirements in the reconfiguration phases (cf. $R_{1}$ and $R_{2}$ in Figure 3). This approach reduces the needed amount of robots. In our example in Figure 3, a conventional heterogeneous system would need 24 devices (10 devices in S, 3+5 additional differently configured devices in cO, and further 6 differently configured devices in Re). With our approach exploiting the reconfiguration capabilities of robots, we only need 10 devices that can be configured appropriately in each phase.

*(C2) Specialization reduces robustness:* For *(D2)*, we propose appropriate self-organization abilities to compensate for failures of different kinds and increase robustness. As homogeneous systems are robust against interferences per definition [49], we propose this characteristic also for multipotent systems. To achieve an autonomous-as-possible execution of user-defined tasks, we need to further endow our robots with self-organization abilities, reducing the number of interactions required between user and ensemble to a minimum.

*(C3) Planning and allocation complexity:* To come by *(D3)*, we reduce the problem of multiagent planning to that of single-agent planning [32] by considering the ensemble as one entity (the collective acts as a single agent). This way, we map the problem of multirobot task allocation (MRTA) [35] from the class of multirobot tasks, multitask robots (MR–MT) to the class of single-robot tasks, multitask robots (SR–MT) [35]. This mapping is possible in multipotent systems when collective algorithms originally designed for homogeneous systems (e.g., swarm robotic algorithms [49,50,51]) implement the actions used for task execution. Actions of individuals do not have to be planned in such algorithms but only to be triggered in the form of local rules that collectively produce a desired emergent effect. Our objective here is to create plans for ScORe missions on ensemble level instead of agent level where possible and use the self-organization abilities of the ensemble to execute these collective tasks. This reduction of complexity facilitates the act of planing for collectives so that plans can be easier calculated and adapted at runtime [32].

*(C4) Proprietary software solutions:* In most current approaches, the capabilities of a robot system are programmed directly against the used hardware (c.f. approaches in References [25,26,52]). To overcome *(D4)*, we propose a declarative approach based on specifying the requirements to fulfill a given robot task. At runtime, the ensemble matches these requirements with the available hardware modules and their basic capabilities. Moreover, the approach also allows to match requirements with combinations of multiple hardware modules. Hence, our objective is to establish a methodology for the abstract definition of requirements for robot tasks and a mechanism that automatically maps them to the (combined) capabilities of available hardware modules. This mechanism and the reasoning of which hardware modules have to be attached to match the requested requirements are the prerequisites for the physical reconfiguration of robots at runtime we want to achieve.

*(C5) Specialized hardware platform:* In the case study, firefighters should reconfigure the hardware composition at runtime on the basis of unpredictable events (e.g., Re in Figure 2). To establish this degree of modularity and to address *(D5)*, we need to introduce individual mechanisms for both the physical and semantic interconnection between hardware elements. Simple hardware elements (e.g., sensors) or even complex systems (e.g., quadrocopters) must be encapsulated within a module containing these mechanisms. Further, every such device has to provide its capabilities and a semantic description of them at runtime. We call this kind of extended hardware self-descriptive devices (SDD). Our objective is to enable the development of SDDs which provide their capabilities in a unified and semantically enriched way to establish physical reconfiguration at runtime without the necessity of modifying the implementation.

Summed up, our reference system architecture for multipotent systems presented in Section 4 is meant to deliver algorithms, technologies, and methodologies to ease the act of programming, employing, and maintaining ensembles. This will enable the handling of versatile ScORe missions by self-organizing, self-reconfiguring, and self-aware robot ensembles, enabled by self-descriptive devices, in a general, standardized way.

## 4. Architecture

**Figure 4:**
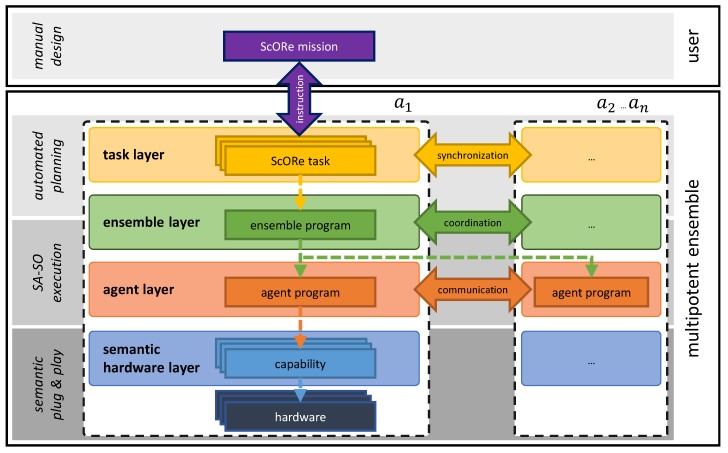
Layered system architecture focusing on robot $a_{1}$ of a multipotent ensemble *A* and its interactions (solid arrows) with the user as well as other robots $\{a_{2},\ldots ,a_{n}\}\in A$. Dashed arrows symbolize an instantiation on the ensemble, agent, and capability layer, valid for executing a ScORe mission. For a specific ScORe mission, the user instructs the multipotent ensemble with typically multiple ScORe tasks have to be solved. Each of those tasks needs one ensemble program that is appropriate for solving the task. Ensemble programs are implemented by the coordinated execution of multiple agent programs across the system. Each of these agent programs specifies the execution order of capabilities it depends on to be locally executable. The executability of capabilities is dependent on a specific agent’s locally available hardware modules. Different background colors depict changes in the technologies we use to handle situations where the ScORe task pattern can be applied to.

**Figure 5:**
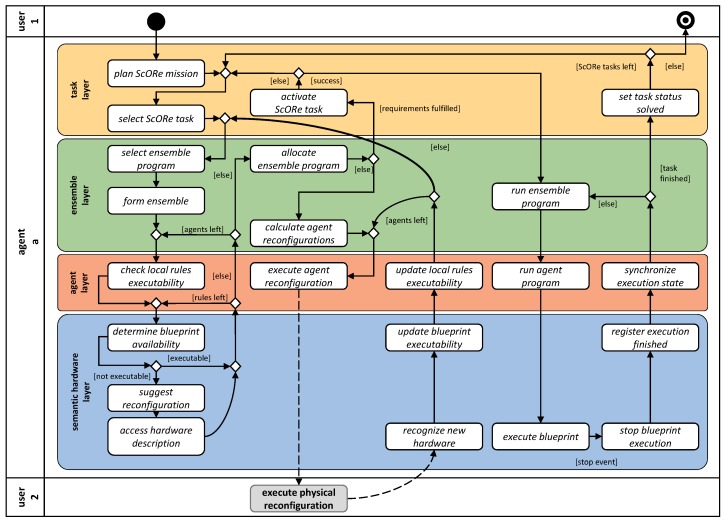
Example of a ScORe mission execution. The activities on task and ensemble layer are executed only on one coordinating robot, while activities on the agent and semantic hardware layer happen on all devices (cf. Figure 4). The interaction with the system’s user is only necessary for the initialization of a ScORe mission (the user is in the role of an initiator: User 1) and the physical reconfiguration (the user is in the role of a reconfigurator: User 2).

To realize our approach for avoiding the drawbacks introduced in Section 1 by achieving our objectives from Section 3, we propose a layered software architecture to be implemented by all robots in the ensemble. This architecture serves as a platform to support necessary functionalities needed to enable a multipotent system. Each layer encapsulates its concepts and algorithms designed to enable self-organization and adaptation (cf. Figure 4). In the following, we introduce our architecture, including a specification of purposes for each of its layers, and give a brief overview of algorithms and mechanisms needed to achieve all goals. This way, we focus on the interplay of all required technologies. Instead of detailed explanations for single algorithms, we want to show the integration of these algorithms in our reference architecture to enable multipotent systems. We refer the reader with interest on the details to the several further reading suggestions we give where they are appropriate. For all algorithms, we assume that robots can communicate without restriction (i.e., through a wireless connection like WiFi or LTE). To enable modularity and adaptability, we model each of our robots with the Jadex Active Components Framework Platform (Jadex) [53] and denote them each as an agent a∈A of our ensemble *A* (cf. Figure 4). In addition offering a communication middleware convenient for many self-organization functionalities, Jadex provides the possibility to dynamically load and unload components from a platform. We exploit this feature to build the core feature of our approach: Dynamically exchanging capabilities of robots that can become highly heterogeneous specialists for dedicated tasks. While our robots are homogeneous at design (i.e., they all have the same knowledge of how to handle specific sensors/actuators in principle), they can become heterogeneous at runtime by (1) their hardware configuration that can be freely manipulated with all available hardware modules, and (2) their capabilities that are a direct result of each of these individual hardware combinations. Because each hardware module brings its implementation with them (i.e., a kind of driver), the software of our robots (i.e., the available capabilities and algorithms) can also become heterogeneous at run-time. To bridge the gap to reality, we develop modular, reconfigurable hardware which can be accessed over its capabilities. Usual hardware elements will be attached to a microcontroller or single-board computer with semantic annotations to describe capabilities. Over a unified interface, these capabilities can be accessed by the agent software. From a software perspective, each of the robots in the ensemble is equal to the others (homogeneity at design time). We show a sketch of the layered architecture of one of these platforms (robot $a_{1}$) in Figure 4. We further depict the required communication regarding interplatform communication with other system participants $\left\{a_{2},\ldots ,a_{n}\right\}$ and communication with the system’s user that introduces the current ScoRe mission in Figure 4. To obtain modularity, communication is restricted to only happening between vertically (intraplatform) and horizontally adjacent layers (interplatform), except for diagonal communication for the coordinated execution of ensemble programs (cf. arrow between $a_{1}$ and $\left\{a_{2},\ldots ,a_{n}\right\}$). In this layered architecture, we assign particular functionalities to each layer, dedicated to solving the user-defined task on a different level of abstraction each. The design also enables the interweaving of classical planning (task and ensemble layer) with SA–SO algorithms and mechanisms (ensemble and agent layer) and combines them with the concept of semantic plug and play (semantic hardware layer and the hardware itself), as indicated by the double arrows on the left hand side of Figure 4. The user is necessarily only involved as the domain expert for the manual design of ScORe missions. In Figure 5, we illustrate the activity of handling a ScORe mission with multipotent systems. Again, also Figure 5 depicts one representative agent $a_{1}$ from our ensemble *A*, including its interactions with the user (in the role of the initiator of a ScORe mission as User 1 and in the role of a reconfigurator as User 2) and other agents (implicitly, e.g., in activities like “allocate ensemble program”). The control flow of Figure 5 illustrates the needed activities that have to be performed on each layer of the multipotent system. In the following, we describe these activities, the interactions between agents on different layers as well as with the system’s user, and other responsibilities of the different layers in detail, each with references in Figure 5.

### 4.1. Task Layer

The task layer serves as a relay between the system’s user and the ensemble. To define the ScORe mission and ensure a user-friendly interface between the system and its user, we propose a domain-specific problem description language for the ScORe system class, based on the concept of hierarchical task networks (HTN) [54]. HTN are especially handy for autonomous systems deployed in real-world environments and enable the design of reusable, parameterizable plans which can be composed for different ScORe missions in a modular fashion [55]. In the way we propose to use them, HTN offer a promising combination of (1) external user control and (2) an appropriate degree of freedom and robustness enabling the executing ensemble for autonomous decisions. While (1) can be achieved by the HTN’s inherent concept of the decomposition of complex tasks into primitive tasks, designed by experts of the domain, (2) is achieved by the proposed SA–SO execution of primitive tasks. In addition to that, an SA–SO execution of primitive tasks will allow the executing ensemble to respond to and compensate unforeseen (i.e., not planned) situations at runtime. This way, we face the fact that by default, HTN planning approaches have only minor uncertainty handling strategies, resulting in extensive replanning, which can be time-consuming [56]: We integrate automated HTN-planning with the paradigm of SA–SO (cf. “plan ScORe mission” in Figure 5). This integration aims at reducing the frequency of replanning by introducing primitive tasks on the collective level, which are not further decomposed for agents, which is the strategy of many current approaches for multirobot systems [21,57,58,59,60]. In situations where the result of an agent’s action does not comply with the estimated effect of a planned primitive task (e.g., a searching agent breaks down, and thus the object of interest is not found in contrast to the planners assumption on the world state), classic planning approaches need to calculate new plans accounting this discrepancy. In our approach, we aim at solving those primitive tasks on collective level by following the intention of aggregate programming [61] (i.e., programming the whole ensemble instead of single agents, cf. Section 4.2) and enable the ensemble to compensate for disturbances during a ScORe task’s execution by itself (cf. *D3* in Section 3).

#### 4.1.1. HTN Definition for ScORe Tasks

**Figure 6:**
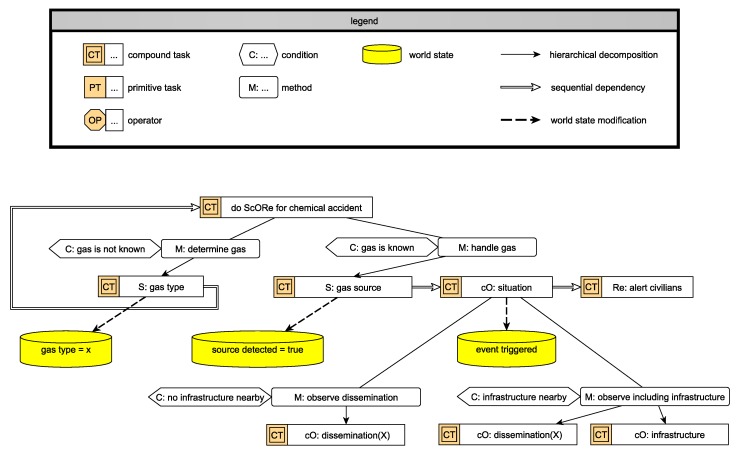
Example of an hierarchical task network for the ScORe mission depicted in Figure 3. The hierarchical decomposition is indicated by black, marked, and directed edges from one task to another. Double-lined, white arrows represent the sequential order of tasks. Dashed arrows depict updates within the world state.

In Figure 6, we showcase an example of an HTN. ScORe missions are represented as complex tasks in the HTN, while the user-defined task is the entry point of the network. The HTN formalizes our running example introduced in Figure 3 taken from our case study of chemical accidents (cf. Section 2). The HTN, as its name suggests, is a network representing tasks as vertexes and their interdependencies as directed edges between them. It offers the possibility to represent hierarchical dependencies (minimal example: A task “transport an object from location A to location B” can be hierarchically decomposed in subordinate Tasks 1–3 with “1: Pick up object at location A”, “2: Move object to location B”, and “3: Drop object at B”) as well as sequential dependencies (the Tasks 1–3 from the previous example have to be executed in sequential order of “1 then 2 then 3”). The idea of hierarchical ordering of the tasks is used to define the necessary steps to solve a user-defined ScORe mission. In such a hierarchy, subordinate tasks have to be solved to solve superordinate tasks, i.e., the subordinate tasks implement the superordinate task. The ScORe mission itself (cf. “do ScORe for chemical accident” in Figure 6) is marked as a compound task, which indicates that it can be decomposed into less complex tasks. The decomposition is done only on the collective level so that planning on agent level is avoided (cf. *C3* in Section 3). As introduced in Section 3, the ScORe mission can be decomposed in one or multiple tasks of types S, cO, or Re. If and how the decomposition of a compound task can be done is defined by associated conditions (if) and methods (how). For each compound task, there may be multiple alternative possibilities for a decomposition, valid under certain conditions that need to be fulfilled in the current situation of the system. To evaluate those conditions, we provide an abstraction of the reality to the system, the so-called world state, modeling the system and its environment with all parameters of relevance. In our example, handling of the ScORe mission is dependent on the condition of whether the most harmful gas is already identified or not. If the most harmful gas is already identified (“M: handle gas” in Figure 6), the typical sequence of S then cO then Re can be applied: Search the gas source, continuously Observe the situation, and React to events by alerting civilians (cf. Figure 6 on the right side). In cases where the most harmful gas is not yet identified, the task is decomposed slightly differently. Prior to the just described other ScORe tasks, the gas type has to be determined (cf. “S: gas type” in Figure 6), which is formulated as an instance of S (cf. Section 4.2 and further for its execution). Accomplishing this task changes the information concerning the gas type in the world state (dashed arrow) and so the subsequent, repeated task “do ScORe for chemical accident” can be decomposed alternatively to apply the default ScORe task pattern. Similarly, the task “cO: situation” can be decomposed in two ways, depending on whether a critical infrastructure is nearby or not. If there is no critical infrastructure nearby, merely the dissemination of the harmful gas has to be continuously observed (“cO: dissemination(X)”). Otherwise, the critical infrastructure has to be observed in a parallel task (“cO: infrastructure”).

#### 4.1.2. ScORe mission Planning, Selection, and Activation

When the user introduces a ScORe mission in the form of an HTN like that given in the previous example, this is broadcasted to the ensemble (no dedicated agent has to be selected by the user). Agents in the ensemble, receiving that mission, will start to analyze it and try to deduce tasks for the ensemble, ensuring that all world-state preconditions hold according to their current model of the world state. vSearching such a valid plan (cf. “plan ScORe mission” in Figure 5) can be done by, e.g., the widely established A* search [55]. The result of that planning consists of ScORe tasks, i.e., such that can directly be executed by the ensemble, e.g., when applying an appropriate SA–SO mechanism. After one robot has achieved a valid decomposition and created a plan, this plan is distributed within the ensemble following the sequential task dependencies retrieved from the plan (cf. “select ScORe task” in Figure 5). This step is asynchronously done in parallel by all agents, and thus multiple (maybe different) decompositions may occur, caused by different world states. The ensemble has to synchronize those planning results and determine one solution to be executed (cf. Figure 4, happening in “activate ScORe task” in Figure 5), e.g., by leader election [62]. ScORe tasks are designed to be parametrizable to enable applicability not only in different situations but also in completely different case studies. For example, cooperatively searching for a specific parameter in a defined area can be parametrized regarding the concerned area, the affected parameter, or a quality requirement (e.g., needed measurement accuracy). Those parameters and requirements must be defined in the ScORe mission and thus be included in its decomposition (i.e., the ScORe tasks). The coordinated execution of ScORe tasks then takes place on the ensemble layer and its subordinate layers, which offer SA–SO mechanisms for solving Search continuously Observe, and React tasks. After solving a ScORe task, the ensemble informs the system’s user about the state of execution concerning the introduced ScORe mission and either decides to autonomously continue its execution if necessary or returns into an idle state. The goal of our approach is not further to decompose plans defined on the collective level and thus directly map these compound task to primitive tasks that can be executed on the collective level. However, in the current state of our developments, we need to still decompose tasks to primitive task precisely defining the individualy needed action of each agent in a specific ensemble, as described in the next section.

### 4.2. Ensemble Layer

Primitive tasks of adapted HTNs (cf. Section 4.1) are formulated as ScORe tasks on a collective level. This design decision raises the need for appropriate actions that have to be defined on the ensemble layer. These actions have to assure the execution of associated ScORe tasks and the production of the expected post-conditions the ScORe mission plan relies on. For every ScORe task type, we provide at least one appropriate action available to solve it on the collective layer, called an ensemble program. An ensemble program encapsulates the logic for the cooperative, coordinated execution of the associated ScORe task. Where possible, we implement those ensemble programs as SA–SO mechanisms, exploiting cases where the interplay of local actions executed on the subordinate agent layer (cf. agent programs in Section 4.3) produces the desired effect as the result of emergence [24] on the ensemble layer. In other words, for each SA–SO mechanism, the ensemble and agent layer have to work in a highly integrated way where structural coordination defined in the ensemble programs (e.g., coalition formation) takes place on the ensemble layer while the execution of the ensemble programs is achieved on the agent layer by local interactions that implement the ensemble program in a self-organized manner.

#### 4.2.1. Ensemble Programs as Implementation for Actions of the HTN

To enable ensemble programs to be suitable for not only one special ScORe task but for a whole class, we design them to be parameterizable, fitting to the also parameterizable task in the HTN (cf. Section 4.1). Many ScORe tasks of class S, e.g., can be handled by the same algorithm where the parameter of interest can be injected—for instance, the source of gas X in gas accidents like in Figure 3, the highest temperature in case of fire accidents, the highest pollen concentration in an environmental monitoring scenario, or any other parameter with a continuous distribution characteristic. As it might not be useful to apply an appropriate SA–SO algorithm (e.g., move a single agent to a predefined location) or not even possible to find one (e.g., in very special Re tasks) we also offer the possibility to fall back on classical multiagent planning [57] to create valid action sequences for solving the ScORe task when necessary. With this strategy, we often exploit the advantage SA–SO algorithms have in planning and executing over current multiagent system implementations: In many cases, actions of agents do not have to be planned explicitly but are deposited as local rules on the agent layer. For example, solving ScORe tasks of class S can be achieved by appropriately adapting and associating a swarm algorithm like the particle swarm optimization (PSO) algorithm [63] to it. Although every agent in the ensemble is working only according to the local rules of the PSO (cf. description in Section 4.3), the collective achieves the objective to find a global optimum as an emergent effect of the cooperation. Other ScORe task classes may be needed for adopting other SA–SO mechanisms, e.g., collective movement with guided boiding [50] in Re, or adapted potential field mechanisms [64] in cO tasks. The goal is to enable the system to exploit situations where multiple different algorithms (ensemble programs) for solving the same ScORe task are available. This decision increases the efficiency and/or robustness of the execution, as each of these algorithms will require different degrees of coordination and autonomy between participating agents and is thus appropriate or not in a given situation. The decision to apply a particular algorithm is made autonomously (cf. “select ensemble program” and “form ensemble” in Figure 5). By evaluating algorithm candidates according to their properties (estimated execution time, communication necessity, etc.) against requirements given by the ScORe task (e.g., in form of a requirement claiming a certain quality level) as well as the current system state (current amount of agents available, environmental conditions), one candidate gets chosen.

**Figure 7:**
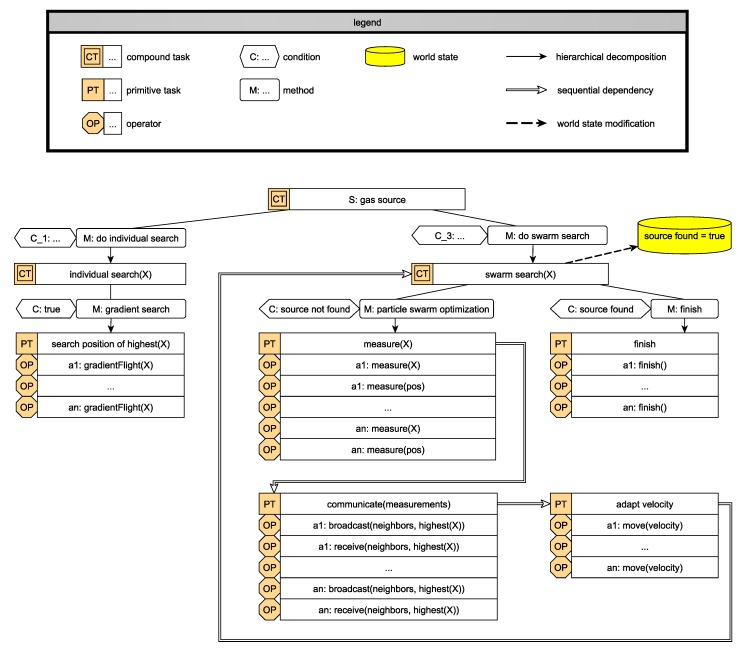
Alternatives for executing an S task depending on environmental conditions defined in the world state given as an HTN. The alternatives require the executability of different local rules, e.g., “gradientFlight(X)” or “move(velocity)”, each depending on specific capabilities that need to be provided by the executing agents (cf. Section 4.4).

Task parameters that influence the decision for specific algorithms can also result from, e.g., the analysis of spatial aspects (the area covered may need certain formations and moving patterns), or time aspects (continuity defined in a cO task may need a frequent substitution of the executing agents). In Figure 7, an example for such alternative possibilities for solving a ScORe task of type S is depicted. Each alternative thus requests a different degree of coordination. Decomposed from the compound task “S: gas source”, the task “swarm search(X)” achieves finding the source of the gas by executing a PSO algorithm. Being an SA–SO algorithm relying on the cooperation of multiple agents, the execution of the PSO is only suitable in situations where there are enough of those agents available and communication among the agents is possible. The SA–SO algorithm itself is expressed as a sequence of primitive tasks in the HTN that have to be executed by each agent participating in it, which we call an agent program (cf. agent layer in Section 4.3). In the case of the adapted PSO, we illustrate in Figure 7, the agent program consists of the repeatedly executed sequence of measuring the parameter of interest “measure(X)”, communicating it to the other agents (“communicate(measurements)”), and adapting the current movement “adapt velocity”. The requirements for executing this sequence can be derived by analyzing each primitive task’s operators (“OP” in Figure 7). These operators describe the concrete actions an agent needs to be able to perform to be capable of solving a primitive task. In Figure 7, e.g., each participating agent *a*_1_–*a_n_* needs to be able to measure the parameter of interest in “measure(X)”, measure its current position in space in “measure(pos)”, broadcast to and receive messages from neighbors in “broadcast(neighbors, highest(X))”, “receive(neighbors, highest(X))”, and move with a given velocity in *myemph(velocity)*. Fewer requirements concerning communication are needed when applying “individual search(X)”, instead, where each agent *a*_1_–*a_n_* is required to be able to perform a sensor-based flight according to measurements of parameter X, i.e., a “gradientFlight(X)”. More information about how agents validate their qualification concerning their current abilities against the task requirements can be found in Section 4.3 and Section 4.4, respectively.

#### 4.2.2. Executing Plans on Ensemble Layer

One responsibility of the ensemble layer is to determine the appropriate mechanism for the system’s current situation according to the tasks’ requirements. This is achieved by forming groups of agents (i.e., task allocation through coalition formation [65]) capable of solving a respective ScORe task with the algorithm best fitting the current situation. As SA–SO algorithms like PSO (and most classically planned solutions) may require the cooperation of multiple agents, coalitions (i.e., subsets of the multipotent ensemble) are formed by the interplay of ensemble layer and agent layer. This way, the ensemble layer of one agent interacts with the agent layer of many other agents (cf. Figure 4). While the one coordinating agent tries to find enough agents to ensure a specific algorithm’s execution, the other agents participate in that finding by evaluating their qualification (i.e., if they meet the defined requirements) for participating in that algorithm (cf. Section 4.3). This split-up of responsibilities reduces the occurrence of problems that otherwise would be caused by a single point of failure as the information on each agent’s qualification is stored locally and not evaluated centrally but distributively. This prevents problems caused by the spontaneous breakdown of agents (when an agent does not respond to the ensemble layer, its qualification can be seen as nonexistent) and parallelizes computationally expensive activities. Like illustrated in Figure 5, “allocate ScORe task” can only be achieved, if all requirements defined in the task are fulfilled. To achieve such a formation of coalitions consisting of appropriately configured agents for given tasks, we integrate a market-based algorithm [66] (cf. “select ensemble program” and “form ensemble” in Figure 5) on the interface between ensemble and agent layer. If an ensemble fulfilling all requirements can be found, the ScORe task can be activated on the task layer (cf. “activate ScORe task” in Figure 5) and executed coordinately (cf. “execute ScORe task” in Figure 5).

#### 4.2.3. Robustness Through Redundancy and Reconfiguration

To achieve robustness and further increase autonomy in the ensemble, the ensemble has to be able to compensate for failures of agents as well as in other hardware (e.g., gas sensors). Therefore, we allow for the existence of multiple ensembles in parallel that work on the same or a different task simultaneously. This can increase performance, e.g., by splitting up the search space for performing a parallel search. To increase robustness, we allow horizontal interplatform coordination on the ensemble layer, e.g., to exchange agents among ensembles when necessary. This strategy can be used to compensate for hard failures (break down of agents) as well as for soft failures (battery depletion). To achieve this, we combine a transactional task execution mechanism with an extended coalition formation algorithm that allows for exchanging agents among ensembles.

Another mechanism is needed when the tasks’ requirements and the system’s configuration do not match but can be adopted appropriately by reconfiguring agents according to their physical composition within the ensemble. This situation can be formulated as an instance of the multiagent resource allocation problem (MARA) [67]. To provide robustness on the ensemble level in such situations, we propose a generalized portfolio approach for solving the MARA [47]. This strategy is used to reconfigure agents within the ensemble. It refines the “calculate agent reconfigurations” activity from Figure 5. Combined with a task allocation algorithm, it maintains the ensembles’ operability. In short, with that approach, we are able to determine resource (SDD) allocations in the ensemble to fulfill all task requirements that have to be fulfilled by agents. To achieve this, we exploit the possibility to decompose the calculation of a solution to the resource allocation problem we already applied very successfully in the energy domain [68]. Consequently, when there is no valid solution to the task allocation problem, the ensemble is reconfigured to ensure a subsequently restarted task allocationand execution of the tasks afterward. This activity needs to be executed in situations like that marked with *R* in Figure 3, where the system needs to be reconfigured according to the changed task requirements.

### 4.3. Agent Layer

An essential part of our approach is agents that can adapt their capabilities to increase their usefulness for the ensemble. Their responsibilities are manifold. To execute ensemble programs, agents in an ensemble have to cooperate. Thus, while structural coordination of those ensembles is handled on ensemble layer, the agent layer is responsible for executing the necessary local rules achieving the self-organized execution of them.

#### 4.3.1. Participating in SA–SO Mechanisms

**Figure 8:**
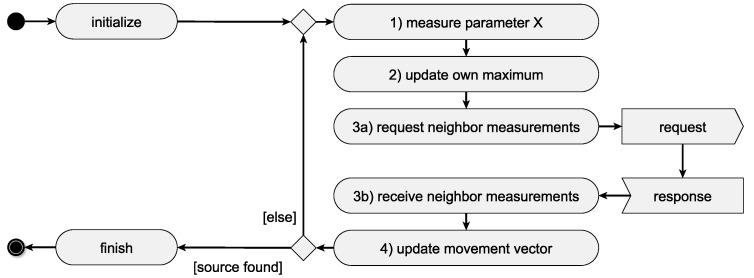
Local rules for executing the particle swarm optimization algorithm [63]. Each participant follows four simple rules: (1) Perform a measurement of the parameter of interest (X) including the location of the measurement. (2) Update the position of the locally found maximum concentration of X, if necessary. (3) Broadcast a request to retrieve all neighbors’ local maxima of X and wait for responses. (4) Adjust the current moving direction according to the weighted average of the local maximum of X, the global maximum of X, and a random component (weights can be adjusted over time).

For every SA–SO algorithm on the ensemble layer (cf. ensemble program in Section 4.2), a local agent program implementing the required behavior as a simple rule-set is known by each agent (e.g., cf. Figure 8 for participating in PSO search, a formation flight [66], or flocking [50]). This knowledge allows the agent to take part in the respective ensemble program. The agent program can also contain communication patterns, defining information exchanges between agents on the agent layer or between the agent layer and the ensemble layer (cf. Figure 4). Communication between agent and ensemble layer is necessary to coordinate collective actions. Messages are mainly sent to inform all participating agents about the starting and ending of collective actions but can also be used to synchronize the ensemble during the execution of an ensemble program (e.g., collective transport [66]). Communication between agents on the agent layer is frequently necessary for the execution in the course of executing an SA–SO mechanism. In the case of the PSO, e.g., it is indispensable for the efficiency of the algorithm that agents can communicate their current measurements and positions of those measurements to requesting agents. In addition to simple participation in ensemble programs, agent programs are subject to a common life-cycle management, e.g., considering the start, end, abortion, or handing over of data in case of an agent substitution in an ensemble or during a ScORe task execution. To increase flexibility and expandability, agent programs can be parametrized similarly to the self-organization algorithms on the ensemble layer. Those parameters are the requirements that are needed for the execution of the agent program. For example, an agent program implementing the search for a gradient of a certain measured value (cf. “X” in “gradientFlight(X)” in Figure 7) can be used for various measurable quantities with continuous distribution, like gas concentration or temperature. Depending on its hardware composition, an agent has the needed capabilities to search for the gradient of a specific quantity (cf., Section 4.4), which in turn qualifies the agent for participating in an ensemble program (cf. “check local rules executability” in Figure 5).

#### 4.3.2. Self-Awareness Supporting Physical Reconfiguration

The determination of currently fulfilled requirements is done by a controlling instance running on every agent, which reacts to changes in the hardware composition and interacts with the semantic hardware layer (see Section 4.4). With this dynamic and autonomous mechanism, changes in the set of an agent’s currently fulfilled requirements will be detected (cf. “update local rules executability” Figure 5) and can thus be respected in future ensemble formations. In combination with the semantic hardware layer, the controller is also able to propose potential local changes in its hardware configuration to better fit an agent program’s requirements (cf. “suggest reconfiguration” in Figure 5), if this is requested by the ensemble layer (cf., during “form ensemble” in Section 4.2). This calculation of reconfiguration suggestions is necessary if an insufficient number of agents is found on the ensemble layer for solving an activated ScORe task (e.g., when switching from S to cO, respectively, from cO to Re in Figure 3), or if the system tries to increase its performance to solve the current ScORe task. In both cases, the local reconfiguration suggestions are calculated based only on local and common knowledge to reduce complexity. Local knowledge involves the agent’s current hardware devices, whereas a common knowledge base is queried about all types of devices that have been registered during the preparation of the ScORe mission. However, the common knowledge base will not contain dynamic information like, e.g., which device is currently mounted to which agent. The agent layer will rely on the semantic hardware layer to determine the executability of capabilities. Reconfiguration suggestions are finally communicated to the ensemble layer, where the information can be aggregated and a decision about actual reconfigurations can be made (cf. “calculate agent reconfigurations” in Figure 5), which involves considering the total cost and the effect of the reconfigurations on the ensemble as a whole. The result passed back to the agent may contain the order to exchange hardware modules (cf. “execute agent reconfiguration” in Figure 5) which can also be performed by the system itself in general (e.g., the need for reconfiguration can be formulated as task itself that is performed by an agent within the system that fulfills all requirements needed to reconfigure other agents) but is performed by the system’s user in the scope of our current research. A detailed description of the full process can be found in Reference [47]. To enable the self-awareness functionalities of agents (e.g., needed for “determine blueprint availability” on capability layer in Figure 5), we designed capabilities as agents within the Jadex Active Components Framework [53] combined with semantically annotated, self-descriptive hardware modules, so-called self-descriptive devices (SDD) [69].

### 4.4. Semantic Hardware Layer

To deduce available capabilities from specific hardware configurations as well as to realize actual execution on real hardware, the semantic hardware layer is implemented as a software adapter to the hardware. It serves as a unified interface for the agent layer to access the current capabilities of the hardware modules attached to a robot and suggest reconfigurations in case an agent program’s requirements cannot be fulfilled. Requirements (cf. Figure 7) define what the robot is supposed to do and must be matched to a set of basic capabilities and, thus, a set of hardware modules. This matching of requirements with actual hardware modules is done by *blueprints* which offer a declarative and semantically enriched methodology to describe composed capabilities and their properties. At runtime, blueprints can be instantiated based on the available hardware and executed. Hence, blueprints are responsible for making the software (i.e., the available capabilities and algorithms) heterogeneous for the robots of an ensemble at runtime.

#### 4.4.1. Requirements and Blueprints

A ScORe mission is triggered by an external user of the system (cf. Figure 4). In this step, the requirements are defined for further task decompositions and matching algorithms, e.g., after the first reconfiguration in Figure 3, the previously identified gas type is required for the subsequent task. This influences the further decomposition of tasks (cf. Figure 6) and is essential for the selection of the right set of hardware to be able to fulfill the task. Depending on the results of the phase “searching gas source & type”, the set of hardware combination and requirements can vary at runtime. Requirements can contain qualitative information (e.g., gas type of the sensor matches precisely with the required gas type), as well as quantitative information (e.g., the minimal accuracy is greater or equal as the required accuracy). During decomposition on different layers, requirements can be added, removed or split.

**Figure 9:**
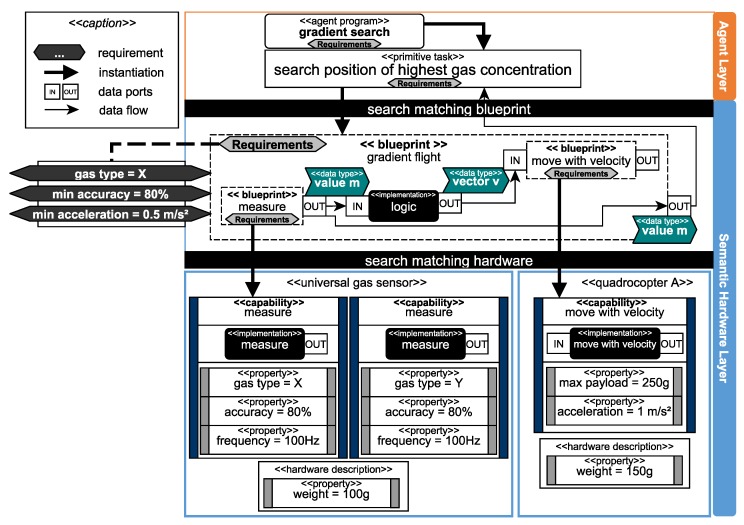
Illustration of an agent program gradient flight, executed on a hardware composition consisting of Quadrocopter A and a universal gas sensor. The blueprint gradient flight resolves the requirements given by the agent layer (see Figure 4) and matches the capabilities offered by the hardware modules dependent to their properties.

Between the specification of a requirement (e.g., “gradient flight” with parameters like the gas type, or the minimal accuracy, cf. Figure 7) and the decomposition into basic capabilities of available hardware modules, a lack of information exists. To eliminate this, we introduced blueprints [70] to describe how to compose individual capabilities to more complex capabilities. Blueprints are a schematic representation of composed capabilities and define how capabilities may be interconnected to more complex capabilities. For example, within the blueprint “gradient flight” (cf. Figure 9), the capability “measure” is combined with the capability “move with velocity” to achieve the composed capability “gradient flight”. The requirements are also divided and allocated to the respective blueprints corresponding to their affiliation, e.g., the gas type is relevant for the measure blueprint.

**Figure 10:**
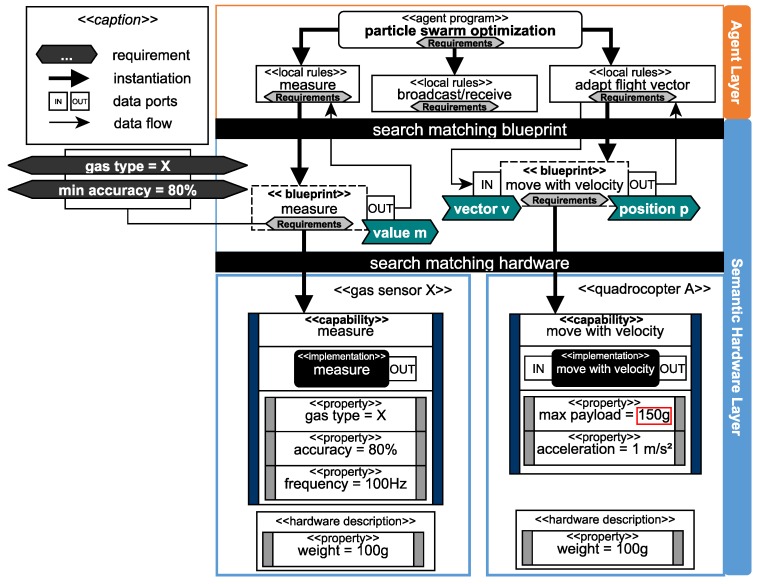
In this example, a particle swarm optimization agent program is split in three different interdependent local rules. The requirements of local rules can be fulfilled by the properties of the self-descriptive device (SDD). However, the local rules cannot be executed due to indirect dependencies between the property max payload = 150 g and the overall weight of 200 g, derived from the hardware description.

Blueprints can contain other blueprints as well as logical implementations (e.g., algorithms to map a value to a vector). They also define the data flow between other blueprints or internal implementations as illustrated in Figure 9. Blueprints have to be instantiated with actual capabilities to make them executable. Hence, every basic as well as every composed capability has a blueprint with a generic description of requirements (e.g., “measure” in Figure 9 and Figure 10). The instance of a blueprint is dependent on the available capabilities of the currently attached hardware modules and their properties (cf. “determine blueprint availability” in Figure 5). For example, the blueprint “measure” inside the blueprint “gradient flight” in Figure 9 can be instantiated with either a capability to measure gas type X or to measure gas type Y.

The data flow between blueprints can also be implemented on a higher level, as it is presented in Figure 10. In this approach, the agent program “particle swarm optimization” needs a high degree of communication between different agents to share the gas measurements and their respective positions where they were performed. The implementation of each agent is reliant on the shared information, so the blueprints are only used to match the requirements with the appropriate set of hardware. This methodology is suitable for nonreal-time critical applications with high dependencies to the intercommunication between agents that can cause high latency but do not suffer from it. In case of a sensor based flight with real-time critical aspects, as shown in Figure 9, the logic is nested within the blueprint to grant a real-time critical execution, as latency must not be introduced in such situations.

#### 4.4.2. Capabilities and Properties

The description of hardware devices is subdivided into capabilities and properties of the hardware component [70]. We define capabilities as executable actions of a hardware device (e.g., “measure” or “move with velocity”) with direct access to the hardware interface. Properties are static information, which are available for a hardware device and serve as a description of geometric dimensions, weight, units, etc. Moreover, properties are used to describe the capabilities of a hardware device (cf. Figure 9). Capabilities are located on an adapter attached to hardware elements like sensors, actuators or combined systems thereof (e.g., quadrocopter). Hardware systems can provide more than one capability (e.g., the universal gas sensor in Figure 9 providing gas type X and Y). The capability is defined by its properties, e.g., the universal gas sensor has two “measure” capabilities with a different quantitative property, which describes the measured gas type.

**Figure 11:**
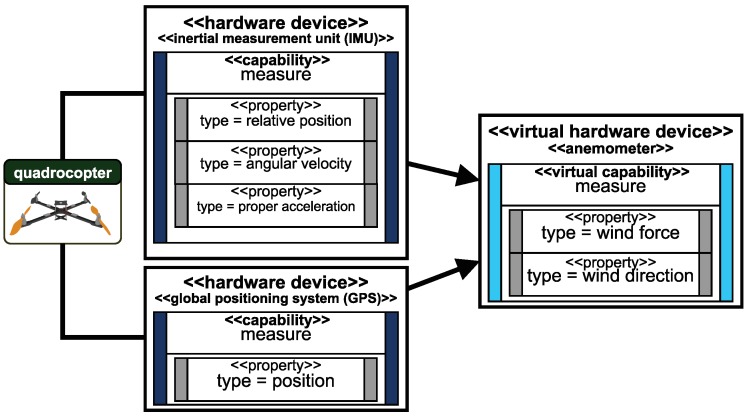
This image illustrates three different types of capabilities which are provided by a quadrocopter. The first capability measure of the IMU demonstrates that a capability can offer different types (e.g., relative position, angular velocity). The GPS instead offers only one capability of the type position. Both have an implementation with direct access to a hardware element. The anemometer instead uses the measurements of the inertial measurement unit (IMU) and GPS to estimate the wind force and direction.

The granularity of capabilities can vary, so a very complex capability (e.g., move with velocity) is based on multiple measurements (e.g., position tracking) and logical parts (e.g., a position controller). Such complex capabilities can be expressed, on the one hand, with blueprints and, on the other hand, as capability, if the hardware is not reconfigurable. The inertial measurement unit (IMU) of a quadrocopter, e.g., provides different measurements but cannot be physically split. Derived capabilities, based on other capabilities as illustrated in Figure 11, are called virtual capabilities. In this example, the capabilities of a quadrocopter are used to realize an anemometer as described in the approach of Palomaki et al. [71]. Both hardware elements (IMU and GPS) are hardwired to a quadrocopter and cannot be reconfigured independently. The benefit of virtual capabilities is that the effort to store and match the correct hardware on resources can be reduced. This is important, as resource management can be a very complex task. We address the problem of resource management concerning capabilities attached to hardware in Reference [47].

Properties specify capabilities as well as hardware elements (hardware description), as illustrated in Figure 10. For the interpretation of properties, we use a common dictionary to define descriptors, such as, e.g., “accuracy”. To ease the creation of properties, further rule sets support the developer, which define that, e.g., the descriptor accuracy must use a value in percent. Properties and capabilities, as well as blueprints, have to be stored in a distributed manner directly on the appropriate hardware for the usage in a multiagent system. Every agent in our layered architecture (see Figure 4) must have access to the actual set of available hardware modules.

Based on the properties of a capability, a blueprint decides if an instantiation is possible (cf. “determine blueprint availbility” in Figure 5). In some cases, implicit dependencies as illustrated in Figure 10 can prevent the instantiation. To instantiate both blueprints in Figure 10, two different hardware modules are needed (“gas sensor X” and “quadrocopter A”). In combination, both elements have an overall weight of 200 g, which exceeds the maximum payload. In this example, the property “payload” from the capability “move with velocity” is set to 150 g. These dependencies among hardware elements can only be checked after the blueprint matching phase when a set of hardware is selected. In the case of the example, a new sensor with less weight or a quadrocopter with a higher payload has to be found to instantiate the blueprint (cf. “suggest reconfiguration” in Figure 5).

### 4.5. Self-Descriptive Devices

The concepts presented in Section 4.4 describe a static view of the system with the goal of an abstract description and usage of available hardware modules. This chapter introduces a middleware for self-descriptive devices (SDD) [70] to fill the gap between the concepts of Section 4.4 and their realization on hardware modules. The idea behind the middleware is to gain access to modular, dynamically coupled hardware modules with decentralized, self-descriptive mechanisms for realizing the access to their capabilities (cf. “recognize new hardware” in Figure 5). Following the idea of the administration asset shell [72], every hardware module is connected to a special software adapter, which can be realized on a single-board computer or a microcontroller. We call this adapter a semantic shell [69]. A semantic shell offers its stored capabilities as well as properties over a unified communication interface. The hardware interface can only be accessed over the corresponding capabilities to ensure a common methodology. Hence, it allows for changing and heterogeneous hardware configurations among the robots of an ensemble during runtime.

In the example from Figure 3, every hardware module (e.g., quadrocopter, gas sensor, camera) will be extended with this semantic shell to be able to provide its semantic description, i.e., its available capabilities and properties at runtime. This composition of a hardware module and the semantic shell is called SDD. For the execution of capabilities defined over multiple SDDs, a dedicated SDD will be selected to host a runtime environment that can also be used for the multiagent system. This dedicated SDD also collects the semantic data of each connected SDD and merges the semantic description to provide an access point for the agent layer. This access point is needed to match the requirements defined at design time with the actual set of capabilities available at runtime. In the example from Figure 9, the quadrocopter as the main component is predestined to host this runtime environment. The schematic view on the combination of SDDs is shown in Figure 16.

Furthermore, the runtime environment is responsible for instantiating and executing blueprints (cf. “execute blueprint” in Figure 5). The execution itself can be asynchronous (e.g., similar to topics in ROS [73]) or synchronous (e.g., similar to the realtime primitives interface [74] the robotics API [75]) to guarantee real-time criteria as mentioned in Figure 9. For example, a quadrocopter from Figure 3 equipped with a gas sensor observes an area. The executed capability “gradient flight” is based on a blueprint which must utilize two different SDDs (see Figure 9). Thus, the measurement of the gas sensor influences the flight direction of the quadrocopter. The physical communication interface between SDDs is realized based on existing technologies (e.g., Ethernet, Ethercat) within a conglomeration of SDDs. If an SDD connects to another, their semantic description will be automatically exchanged. The implementation of a vertical prototype is presented in Reference [69] with mechanisms of the semantic web [76].

## 5. Proof of Concept and Preliminary Results

Our preliminary results include evaluations in three main categories that are of great relevance for our approach: (1) The feasibility of executing ScORe missions in the real world involving real robots, (2) the appropriate functionality and the interplay of all SA–SO algorithms necessary to realize the idea of multipotent systems, and (3) the practicability of physical reconfigurations concerning the hardware configuration of our robots. In the following, we present our results in these three areas and thus demonstrate the general feasibility of using multipotent ensembles to accomplish real-world tasks defined with the ScORe task pattern. For all of our results so far, we also state how they integrate in our layered architecture that we introduced in Section 4.

### 5.1. Executing ScORe Missions in the Context of Environmental Measurement

In the context of environmental monitoring, we identified that the investigation in conditions of the atmospheric boundary layer (the layer of air closest to the ground) that geographic researchers perform on a regularly basis [20] can be formulated as an instance of our ScORe task pattern. Often, certain conditions in the boundary layer have to be continuously observed to enrich, e.g., meteorological climate models with appropriate real-world data.

Our goal during two real-world experiments (called ScaleX 2015 [20] and ScaleX 2016 [77,78]), therefore, was to verify the feasibility of using mobile robots for ScORe missions in the context of environmental measurements. As we were focusing on the feasibility of accomplishing a ScORe mission in general, we performed all experiments during ScaleX 2015 and ScaleX 2016 with a fixed set of capabilities for all involved robots and neglected the ability to reconfigure hardware configurations. Throughout the first experiment (ScaleX 2015) [20], only ensembles consisting of multiple semi-autonomous controlled robots were used. The high effort for maintaining operability during hourly performed experiments clearly demonstrated the great need for autonomous control and coordination for mobile robot ensembles.

**Figure 12:**
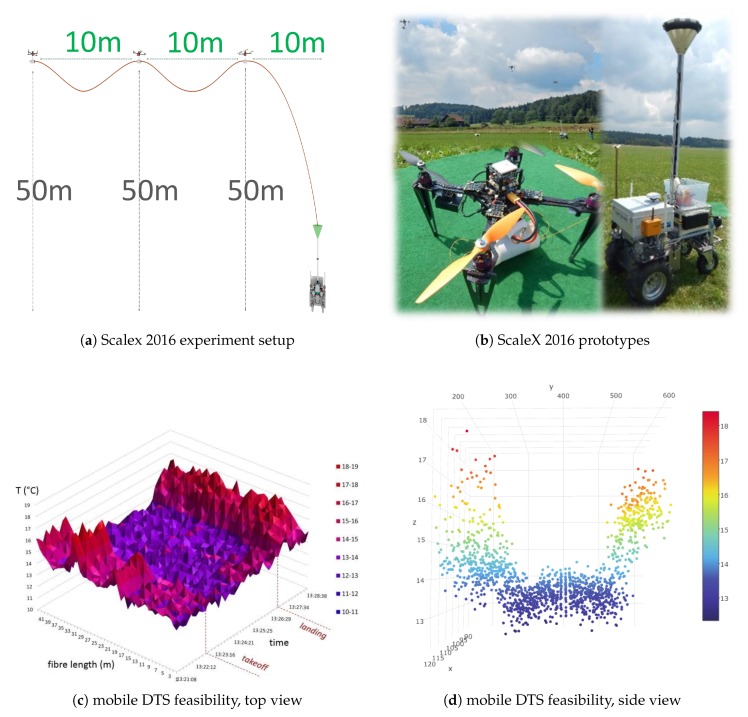
(**a**) shows a sketch of the experimental setup with four heterogeneous robots. A ground robot carries the distributed temperature sensing (DTS) evaluation unit where the fiber cable is attached. Three quadrocopters cooperatively elevate the fiber cable up to a height of 50 m, distributing it horizontally over 30 m. (**b**) shows the mobile robots that we used during the experiment, including the necessary hardware installations to enable the cooperative transport of the fiber cable. (**c**) illustrates measurements achieved during one of the experiment flights. Different temperatures are depicted by different colors (red indicates warm, blue cold) as well as different values of z. Values of x describe the position along the fiber cable a measurement was made at. Values of y show the development of measurements achieved during the flight (given in time UTC+2). (**d**) shows another perspective on the results with the starting position to the left and the landing position to the right, clearly depicting the cooling during flight in 50 m height (red indicates warm, blue cold).

We tackled this issue in the follow-up experiment during ScaleX 2016 by introducing autonomous robot control as well as ensemble coordination. Therefore, we used a first vertical software prototype of our layered software architecture (cf. Section 4) with an implementation of the ensemble, agent, and semantic hardware layer. We analyzed the temperature profiles in the boundary layer (the atmospheric layer closest to the ground) during daytime. Our experiment setup included the coordinated and cooperative transport of a large-scale temperature sensing unit (a fiberglass cable) to achieve temperature profiles with the distributed temperature sensing (DTS) [79] technology. With the DTS technology, temperature measurements can be made within every meter of the carried fiberglass cable [80]. The cable was carried cooperatively in a line-like formation along a defined route in the experiment area in the height of approximately 50 m (cf. Figure 12a), which introduced physical constraints that requested a high grade of coordination during the execution of the experiment. The task was performed autonomously by a heterogeneous ensemble (coordinated within the ensemble layer), consisting of multiple quadrocopters as well as a mobile ground robot (cf. Figure 12b) that were each controlled by their individual agent layer. Task allocation, assignment, and the coordinated execution were performed in an SA–SO fashion, respecting the agents’ different available capabilities (quadrocopters can move to positions higher than 1 meter, mobile ground robots cannot) and the tasks’ required capabilities (some tasks needed the ability to reach up to 50 m, others to carry the 7–8 kg heavy evaluation unit). To enable the robots to match their (fixed) set of available capabilities to the tasks’ requirements, a first version of the semantic descriptions within the semantic hardware layer was used. A sketch of the experiment setup, including the results achieved in this experiment, can be found in Figure 12a–d. In addition to the original intention for this experiment, which was to demonstrate the benefits of our architecture when integrating different platforms equipped with different sensors in an ensemble, we were also able to show the feasibility of large-scale temperature measurement achieved by cooperative transport. Figure 12c,d depicts the measurements achieved with the DTS during one flight. In Figure 12c, the course of changes in temperature measurements along the DTS fiber maps the robots’ original path of movement. The experiment was performed shortly after midday where temperature gradients in the lower boundary layer follow the classic shape (warmer at the ground and the higher, the cooler). This way, the time of aerial measurements between takeoff and landing can be clearly identified through lower temperature measurements of approximately 13.0 °C and lower in the time during 13:23:16 o’clock (UTC+2) and 13:27:34 o’clock (UTC+2) compared to temperatures above 17 °C on the ground level. One can also see this effect in Figure 12d, where we plot the measurements of the same experiment in an additional side view that emphasizes the contrast between temperatures at ground level (red, orange, yellow, and green color) and aerial temperature measurements (blue colored). Similar coordinated flight patterns can be used in Re tasks of our ScORe task pattern, e.g., when the ensemble has to inform civilians in a widely distributed area that also requests for a coordinated line-like formation.

**Figure 13:**
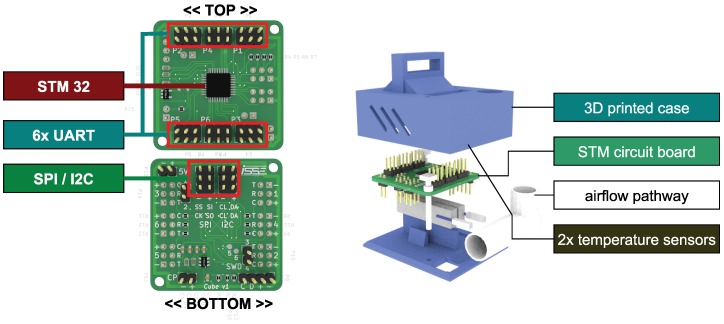
On the left side, the self-developed break out board for an STM microcontroller is illustrated. The STM circuit board supports up to six UART, one SPI, and I2C interfaces to connect sensors or actuators. The prototype on the right is specially designed for environmental measurements. On the STM board are two TEMOD boars with temperature sensors connected, which are placed in an airflow pathway. The prototype is mounted under the rotors to use the downwash for optimal airflow.

**Hardware Setup:** We built an SDD prototype, especially designed for environmental measurements, with a microcontroller on a self-developed break out board (see Figure 13).

The custom board supports SPI, I2C, and UART for the communication with attached sensors or actuators (see Figure 16). At least two UART connections are reserved for the communication between SDDs. The STM has enough performance for small computational tasks and uses the limited internal persistent storage to deliver its self-descriptive information. The prototype used the internal storage for semantic annotations as well as for calibration measurements to calculate calibrated measurements at runtime to reduce the post-processing effort. We used up to four quadrocopters with frames from *rOsewhite* [81] integrated with an Autoquad flight controller [82]. Equipped with an Odroid XU4 [83] each that was connected via UART to the flight controller, we thus formed a quadrocopter encapsulating SDD. The ground robot was built upon an Innok Heros [84] that carried the DTS measurement unit (a Oryx DTS measurement device [79]). To carry the DTS, we attached specialized flexible modules to the quadrocopters as well as to the ground unit to avoid damage done to the glass fiber (cf. Figure 12b).

**Conclusion:** Summed up, we proofed our concept of controlling robots with our layered software architecture combined with SA–SO mechanisms for coordination, self-awareness, coalition formation, leader election, and cooperative execution [66]. We further demonstrated that in general, ScORe missions can be autonomously performed by autonomous ensembles of robots. The experiment also clearly exposed the need for more flexibility concerning a robot’s hardware equipment to be able to flexibly adapt, e.g., the measuring capability when this is necessary to further investigate meteorological phenomena. In addition to that, we showed the feasibility of airborne, mobile measurements achieved with DTS technology. For the verification of the taken DTS measurements, we used the prototype from Figure 13 with two calibrated temperature sensors on each quadrocopter.

### 5.2. Integrating SA–SO Algorithms to Realize Multipotent Ensembles

In addition to our outdoor experiments and to validate the applicability of our SA–SO algorithms, we frequently perform indoor as well as simulated experiments. The experiments described in the following involve multiple SA–SO algorithms working in a highly integrated manner. This is necessary to realize multipotent systems like we describe them in our reference architecture Section 4.

#### 5.2.1. Experiments in a Real-World Environment

**Figure 14:**
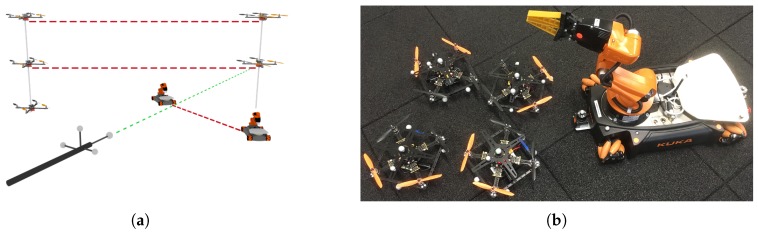
(**a**) Illustration of our test scenario with a magic wand controlling the ensemble via gestures. The position, as well as the formation, can be selected with gestures. (**b**) The heterogeneous ensemble of mobile robots used for indoor experiments, demonstrated, e.g., during FAS*2016 [66]. Each robot is marked with a unique pattern of reflective spheres, making them recognizable for the VICON [86] indoor tracking system used for positioning. For further illustration, a video of our experiment is available online [85].

To evaluate and demonstrate the integration of basic ensemble algorithms for coordination and self-awareness purposes with our robot hardware controllers, we designed an experiment for FAS*2016 [66,85]. The experiment involved a heterogeneous ensemble consisting of mobile ground robots as well as multiple quadrocopters with different capabilities (cf. Figure 14). The multipotent system reasoned from tasks defined on the collective level in a self-aware manner, autonomously formed dynamical coalitions for those tasks, and executed them in a coordinated fashion. The experiment also aimed at an intuitive way to define variable user task at runtime. The user can select the formation, the number of platforms, and the position of the formation. If the position is too near to the ground for a stable flight, the task requires not only the capability of flying but also the driving capability provided by the mobile ground robot. We also defined an area where a ground robot, as well as a quadrocopter, can be used. In this case, the ensemble layer calculates the minimal reconfiguration time for all mobile devices. Depending on the requirements defined by the user, the robots were able to form different ensembles to match those requirements (cf. Figure 14a), i.e., achieved an autonomous adaptation on the ensemble level.

**Hardware setup:** For the indoor experiment, we used an external optical tracking system (VICON [86]) that can identify patterns of reflective spheres and track their position with high accuracy. The tracking system is used to determine the position of every mobile robot in the experimental area. To distinguish the mobile robots, we used a unique asymmetrical pattern of these reflective spheres. Therefore, we expanded the frame of the quadrocopter as illustrated in Figure 14. As a mobile ground unit, we used a KUKA youBot [87]. To define the user tasks, we used a “magic wand” with gesture recognition mechanisms. The magic wand is a simple wood stick also attached with a unique set of reflective spheres. The orientation of the stick, as well as its position, is used to define gestures and change the user task in our layered architecture (Figure 4).

**Conclusion:** The experiment demonstrated the feasibility of a dynamic instruction of ensembles with an intuitive device. It also showed the ability of an ensemble designed according to our reference architecture to autonomously adapt its configuration on the ensemble level. The experiment also showed the need for flexibility concerning capabilities provided by robots. It thus motivated us in our progress of separating those capabilities from robots and towards making it possible to freely combine them at runtime, which we focused on in the subsequent experiments.

#### 5.2.2. Experiments in a Simulation Environment

**Figure 15:**
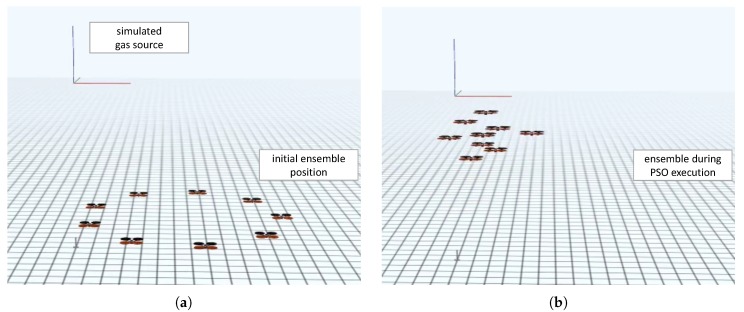

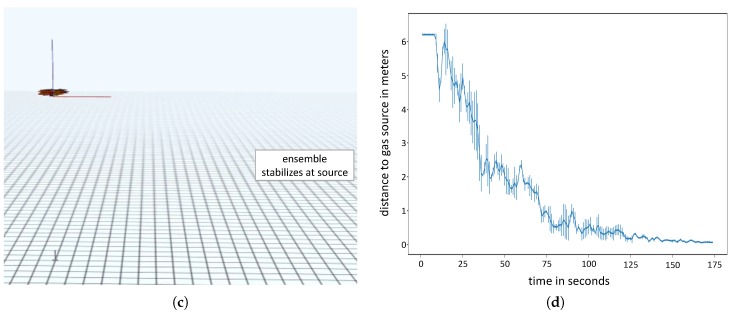
(**a**) shows the system previous to execution with the gas source indicated as a coordinate system (top left) and all quadcopters in their initial circle formation. In (**b**), all quadcopters start to execute the local rules of the particle swarm optimization algorithm (PSO) to approach the gas source cooperatively. (**c**) indicates the state where the gas source is found by the ensemble (no collision avoidance is respected in this simulation run). (**d**) shows the average distance of the quadcopters to the gas source in meters (during one execution of the PSO), including the standard deviation as error bars, continuously reduced with the time passed.

We achieved further results concerning the interplay of SA-0SO algorithms that are necessary to realize multipotent systems in simulation. These results concern parameterizable swarm algorithms for the ScORe task pattern. For all of our simulated experiments, we used a robotics control framework [75], which we also use for controlling our real robots. This way, we reduced the width of the reality gap to a minimum which helped us to migrate our findings from simulation to reality. As an example of an algorithm, we can use for S tasks in the ScORe task pattern we evaluated the particle swarm optimization algorithm (PSO). Figure 15 shows the progress of executing PSO in our simulation environment. The robots need to be able to freely move in 3-dimensional space (flying) as well as measuring the parameter of interest. Agents use their self-awareness abilities to determine their qualification for the task. Measurements are propagated in the ensemble, and the motion vector is adjusted according to the PSO rules (information on the currently measured maximum concentration of the parameter, the local concentration at an agent’s current position, and a specific random component to avoid local optima, cf. Figure 8). This way, agents that cooperate on agent layer by executing the appropriate PSO-specific agent program collectively execute the PSO-specific ensemble program that is coordinated on the ensemble layer. The success of the execution can be seen in Figure 15d. While the average distance to the simulated gas source (cf. Figure 15a) is initially on a relatively high value of approximately 6 m, it is steadily reduced during the execution of the algorithm. After an execution time of approximately 150 s, the source of the gas is detected by the ensemble (cf. Figure 15c), which one can also see in the reduced average distance to the gas source as well as the distribution of the ensemble’s robots, which no longer varies much (cf. Figure 15d). This leads to the correct execution of an S task on the task layer, requiring only local interactions. In addition to the PSO for an S task, we also realized prototypes for other ScORe tasks that promise the successful execution of cO (potential field algorithm) and Re tasks (guided boiding).

**Conclusions:** Our simulated experiments show the feasibility of implementing swarm algorithms (like PSO and the potential field method) within our reference architecture and using them to solve certain tasks of our ScORe task pattern. With the following experiments, we aim at further investigating the performance of those swarm algorithms in comparison to classic (planned) approaches concerning solution quality and consumed time.

### 5.3. Practicability of Physical Reconfigurations with Modular Sensor Systems

Our layered architecture in Figure 4 is based on reactive systems with changing requirements as well as varying capabilities at runtime addressing heterogeneous and reconfigurable ensembles. To demonstrate this reconfiguration and flexibility, we developed modular hardware devices with self-descriptive mechanisms which can be exchanged at runtime. Therefore, we attached a single-board computer to different sensor types surrounded by a 3D-printed case with magnetic connectors and a plug connection.

**Hardware Setup:** To deploy the mechanisms, described in Section 4.5 on modular, self-descriptive hardware elements, several aspects have to be recognized. The requirements for the realization of modular hardware devices resulting from those specifications are:

- Physical interfaces to connect hardware, at least I2C, SPI, and UART;
- a common communication interface (e.g., Ethernet) with at least two IOs for daisy chaining;
- small form factor for the usage in combination also with a quadrocopter;
- sufficient performance for the scope of functions (e.g., to provide a runtime environment);
- persistent storage to store properties, capabilities and measured values at runtime.

**Figure 16:**
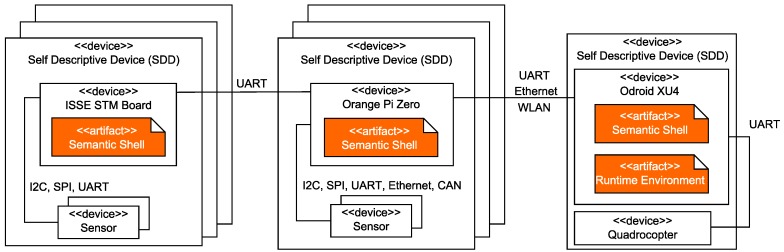
The figure illustrates possible combinations of self-descriptive devices (SDDs) with one quadrocopter and multiple sensors. The quadrocopter is connected to a single-board computer (Odroid XU4) via UART. The sensors are either connected to a self-developed microcontroller (ISSE STM Board, see Figure 13) or to a single-board computer (Orange Pi Zero) over different communication interfaces. The semantic shell (see Section 4.5) is deployed on these devices and serves as a unified controller between them.

As a platform for the semantic shell from Section 4.5 without an additional runtime environment (cf. Figure 16), we used the Orange Pi Zero [88] with an ARM Cortex A7 and 512 MB DDR Ram for the deployment of our concept (see Figure 16) attached on two different distance sensors (ultrasonic and laser range) and two different temperature sensors. The Orange Pi fulfills the interface requirements and offers Ethernet and WLAN additionally. The form factor is, in comparison to other single-board computers, very small (48 mm × 46 mm), with sufficient performance to store and execute capabilities of the attached sensors. Further, a micro SD card with up to 32 GB can be used as persistent storage.

**Figure 17:**
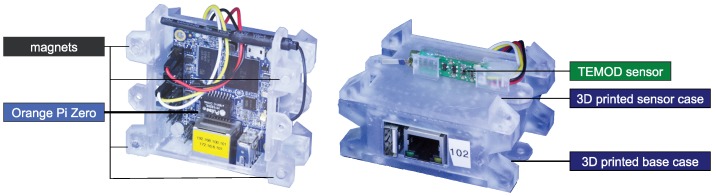
The 3D-printed base case for the Orange Pi has four magnets and can be connected to an also 3D-printed sensor case or to a quadrocopter adapter. As a sensor, a TEMOD [89] board with a PT 1000 temperature sensor is connected to the Orange Pi via an I2C interface.

**Figure 18:**
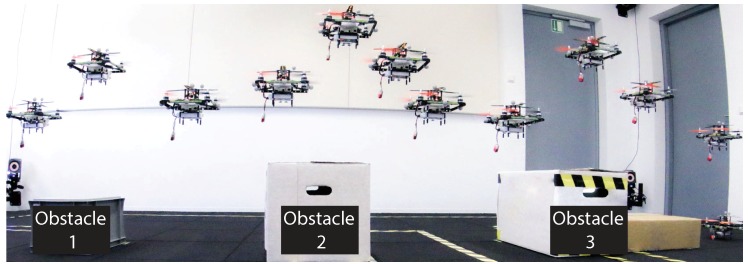
Time lapse recording of a quadcopter equipped with an ultrasonic distance sensor (SR-04) and obstacles on the ground for the sensor-based flight. An external optical tracking system is used to validate the height and as reliable position control for the quadrocopter.

To mount the Orange Pi to the platform, a self-developed quadrocopter, a stackable 3D-printed case with magnetic connectors was designed (see Figure 17) to enable a simple reconfiguration at runtime. To ensure that the quadrocopter will not lose the stack in flight, the case can also be bolted between the magnet pairs for safety purposes. The quadrocopter also has capabilities which can be executed and stored, so the implementation of a semantic shell (see Figure 16) is also deployed on an attached single-board computer. Next to the implementation of a semantic shell, this single-board computer is also responsible as an access point for the agents within our layered architecture. To establish enough performance, we used an Odroid XU4 [83] with an Exynos5422 octa-core ARM processor which is connected via UART to our quadrocopter device. The communication between the flight controller and the Odroid is realized via UART, also with a self-designed expansion board. We used two different quadrocopter frames from *rOsewhite* [81] with an Autoquad flight controller [82] for our prototypes. With the developed system, it is possible to use values of a sensor SDD to influence the behavior of actuators like a quadcopter SDD. Figure 18 shows the flight of a quadrocopter that adjusts its height according to the measured distance to the ground with a distance sensor SDD. The logic of this sensor-based flight is predefined in a blueprint (see Section 4.4.1) and can be mapped with capabilities of attached SDDs at runtime. As a test setup, a quadcopter SDD equipped with an ultra sonic distance sensor SDD flew along a specified route with obstacles. This procedure has been repeated with a laser distance sensor instead of the ultra sonic with qualitative differences, e.g., reaction time, accuracy, and different noise level to estimate how these values will affect the sensor-based flight. For the navigation of the route, an indoor tracking system was used, which also recorded the exact position of the quadcopter. To validate the suggestion of SDDs, the capability “sensor-based flight” can be executed with distance sensor SDDs as well as with temperature sensor SDDs, depending on the task requirements. The sensor-based temperature flight is comparable to a kind of thermometer. If the measured temperature increases or decreases, the distance of the quadcopter to the ground matches the change.

By merging the self-descriptions of each SDD to a common knowlege base, it is possible to generate additional information for the overall system. The total weights, power consumptions, and power reserves are known for each SDD, so it is possible to calculate the average flight time of a quadcopter.

**Conclusions:** The developed SDDs can store its self-description with common interfaces to access simple sensor values (e.g., distance sensor) or even complex execution instructions for actuators (e.g., quadrocopter). We demonstrated that blueprints can be used to combine different capabilities, deployed on different hardware devices, to a more complex capability (e.g., “sensor-based flight”). The capabilities and properties can be merged to a common knowledge base and be accessed by the agent to execute a task if the requirements can be fulfilled with the corresponding capabilities of the SDDs. The estimation of the flight time influences the executability of the capability “fly to position”, so if the estimated trajectory will take too long, the system sends a failure message and a reconfiguration is necessary. A more detailed description of the used sensors, the distributed storage of capabilities and properties, and resource allocation mechanisms can be found in the following three papers: Reference [69] (concrete implementation of a distributed knowledge base with techniques of the semantic web), Reference [70] (matching between requirements and capabilities), and References [47] (resource allocation and estimation of possible configurations with a predefined set of hardware). The generation of reconfiguration suggestions within a distributed knowledge base for an easy usage of our architecture is in scope of future work.

## 6. Related Work

The following sections investigate related work in relevant research fields, emphasizing potentials as well as limitations in the context of ScORe missions. The first two sections illustrate the state-of-the-art considering task definition, planning, scheduling, allocation, and execution. These also include existing approaches from the field of self-adaptation and self-organization. To motivate our vision of self-descriptive devices, the third section investigates existing work concerning knowledge representation and semantic hardware description. The final section gives an overview of current and past projects within the scope of ScORe missions. For means of simplicity, we refer to variable nomenclature found in the literature (e.g., robot, swarm member, individual) solely as agent in the following.

### 6.1. Task Definition and Planning

Employing autonomous, intelligent, and self-aware systems in real-world environments increases the need for efficient (online) planning. In the following, the analysis of the current state-of-the-art in this domain demonstrates issues that typically occur when trying to apply them to ScORe missions. Our approach tackles these issues by combining traditional (well-explored) planning techniques with the technique of self-organization.

Planning focuses on what has to be done to reach a goal state and in which order, i.e., which action sequence leads from an initial system state to the goal state [90]. To appropriately describe different states and describe possible solutions to solve the planning problem, an adequate problem description language is essential. The Planning Domain Definition Language (PDDL3 [91]) is an established technology for doing this. It enables the description of so-called “worlds” (or domains) with possible actions and their effects. We adopt this planning language to cope with the concepts needed for ScORe missions. This requires appropriate environment state abstractions as well as possibilities to define actions in the scope of ensembles. To find suitable plans for adequately formulated problems, state-space exploration [92] is the standard in many planning systems, enabling autonomous search for a transformation of the initial into a goal state. Search approaches either follow the paradigm of forward- or backward-search (i.e., progression or regression) in state space [93], or merge them for bidirectional search (e.g., Reference [94]). In order to reduce the complexity of finding a valid plan, forward-search approaches often use heuristics (e.g., task decomposition in hierarchical task networks (HTN) [34]) as well as efficient data structures (e.g., planning graphs in Graphplan [95]). This can make the planning problem solvable in polynomial time [90]. Other approaches use satisfiability (e.g., SATplan [96]), combine constraint satisfaction programming (CSP) with planning graphs (e.g., GP-CSP [97]), or work with partially ordered plans (e.g., RePOP [98]) to search the space of plans instead of the space of states. The planning problem gets more difficult to solve when a realistic environment is assumed, where the effect of an action on the environment is uncertain [99]. To tackle this issue, developments from the online [56] and agent [100] planning community were developed [59]. Instead of planning on the basis of complete state-space knowledge, agents plan on their belief-state [101]. For this purpose, modeling approaches were proposed, like the widely used BDI model [102] or MAPE-K model [103]. If the execution of plans also needs the cooperation of different agents, planning for distributed execution becomes indispensable [104]. As planning methodologies are mostly implemented for single agents [105], traditional planning approaches must be adapted, when dealing with distributed systems consisting of multiple agents (MAS). Centralized approaches generate multiagent plans, i.e., plans containing the necessary actions of all participating agents [106,107]. There are also approaches for interwoven execution and plan formation in MAS [21,108]. The planning approach used in our approach makes use of these results and adopts them by combining them with techniques using the self-organization paradigm. We assume that this reduces complexity as uncertainties do not have to be considered during planning but can be absorbed during execution by appropriate self-organization algorithms (avoiding time-intense re-planning).

If planning is to be achieved in a completely decentralized fashion, a need for guidance or policies for each agent arises in order to know which actions must be performed to cooperatively reach a goal state [56]. This is known as the multiagent plan coordination problem [55,57,60], where agents first create plans locally and then merge them into a global one. There are also approaches that map the classic solutions of centralized planning onto agent-based decentralized versions (e.g., partial-order planning in distributed HTN [109]). In all cases, planning in multiagent environments needs coordination between agents [21], i.e., planning of individual actions and multiagent coordination must be done together [104]. All multiagent planning techniques have in common that plans contain actions for every single agent of the system. In cases where one agent’s action produces an unforeseen effect, most parts of the multiagent plan may need to be adjusted. As this is very likely in a real-world scenario most ScORe missions are located in, there is the need of a further abstraction in planning, as we plan it with our approach by combining planning on the ensemble level with self-organized execution. MAS cooperation and coordination can be considered as scheduling problems [35], in which previous work is presented in detail in the next section. Current work is often focused on developing comprehensive frameworks like NASA’s *Europa* [28,110], *Plexil* [111,112], or *OpenSPIFe* (used in multiple Mars missions and currently on the International Space Station *ISS*) [113], which offer quick access to planning technologies for users.

A different approach to instructing a collective in an abstract way is proposed in the Protelis Language [61]. This programming language considers the collective of agents as a computational field that can be programmed as an aggregate. A similar idea is proposed in Reference [114], aiming to develop a novel programming language to easily script swarm behavior. These approaches intend to simplify the instruction interface for swarm behavior but do not intend to reduce the planning complexity or integrate the idea of reallocating capabilities, which we intend to do in our approach.

We see high potential in creating a new methodology of MAS planning for real-world environments, by combining classic planning techniques with the self-organization paradigm to reduce complexity on the planning level. Because uncertainties can be handled on the self-organization-layer, they do not have to be considered during planning, which enables prior plan calculation even for uncertain environments. Additionally, all approaches up to now do not exploit the potential arising from the ability to reallocate the agents’ capabilities. Our approach tackles this issue with self-organization principles, which results in much more flexible and adaptable solutions as the current state of the art offers, enabling ScORe mission planning and execution despite possible uncertainties.

### 6.2. Task Scheduling, Allocation, and Execution

In contrast to task planning, task scheduling and allocation concentrates on when and by whom actions should be performed [90]. This decision is often to be made consistent with resource or time constraints [115]. Because the (re-)allocation of tasks (ScORe tasks) and resources (agent capabilities) is of central importance in our approach, other work tackling these problems is investigated in the following. In general, tasks may need to be performed at a certain time while the total make-span, i.e., the time needed to reach the goal state, has to be minimized. This problem stems from the field of operations research [116] and is called the job-shop scheduling problem (JSP) [117]. When considering realistic settings, where scheduled tasks also entail certain resource demands (like processing power, knowledge, capability, information, or expertise [118]), the complexity of scheduling becomes NP-hard [90]. To deal with this difficulty, heuristics are often used (e.g., minimum slack [119], ant system [120]). Other methods increase the flexibility of resource schedules [121] or map scheduling problems to constraint CSOPs to exploit (resource-) constraint propagation [122] to increase performance. Within our approach, the use of these techniques is of high relevance, as the scheduling problem has to be solved frequently on the agent level (to generate appropriate agent hardware configurations) as well as on the ensemble level (to generate appropriate ensemble configurations). To further reduce the complexity of the resource scheduling problem, planning and scheduling can be performed simultaneously in order to already include resource constraints during the creation of partial-order plans [123]. An HTN planner (as mentioned in the previous section) that is also able to plan resources is *O-Plan* [124]. State-space search planners accounting for resource planning are *Sapa* [125] and the heuristic approaches of Reference [126]. When mapping the scheduling problem to robotics, many approaches can be categorized as an instance of the multirobot task-assignment problem (MRTA problem). That is, to decide an allocation of tasks to robots either instantaneously (IA, i.e., task assignment) or time-extended (TA, i.e., task scheduling) where each agent may be able to deal with multiple tasks (MT) or just a single one (ST) and tasks may need exactly one robot (SR) or multiple (MR) to be accomplished, i.e., a classification can be MR–MT–TA or any other combination [35]. Our approach ultimately focuses on the complex problems of this last combination, while simpler combinations are analyzed during the iterative development process of creating the pursued system architecture. The MRTA problem can be further described by analyzing the agents’ abilities: Are they heterogeneous or homogeneous, i.e., do all of them have the same abilities or do they differ [127]? For both possibilities, there exists an abundance of literature on how to solve the MRTA. Heterogeneous approaches often use market-based mechanisms (e.g. References, [128,129]) based on strict communication protocols (such as the contract net protocol [130]) when direct interaction between robots/agents is possible. Other approaches use mixed-integer linear programming [36], stochastic gradients [131], or even scripted behavior [23]. Homogeneous approaches [50,51] are well-established in the swarm robotics community, where robots all have the same, often very limited, abilities [132]. On the one hand, solutions to the MRTA can be determined centrally, i.e., one central agent calculates the assignment for all other agents [133]. On the other hand, solutions can be calculated in a decentralized manner [134]. Decentralized solutions tend to only be suboptimal [127], which still can be sufficient [68]. Task allocation problems can also be solved through coalition formation [65,135]. Current approaches have in common that they do not consider the possibility of reallocating capabilities to agents, which we already tackled in our TRANSFORMRS approach [47] to enable more flexibility in its usage.

Tasks themselves can either have a simple or a complex nature, i.e., can be directly assigned to dedicated robots in the first case or, in the second case, can only be assigned to a group of robots where they subsequently must be further decomposed [136]. As well as the initial planning, the scheduling and allocation of tasks is also indispensable to the revision of previous decisions during execution and for the adaptation to changed conditions [137]. The approach of Partial Global Planning [138] therefore combines task decomposition, planning, scheduling, communication, allocation, and reallocation. Following the same idea, the Continuous Planning and Execution Framework [139] also offers functionalities to repair existing plans and allocations in case of unexpected situations in an unpredictable environment. An aspect where those frameworks differ from our approach is that they neglect the reallocation of agents’ capabilities. In our approach, we avoid in-depth planning of agents’ actions as it is typically done in these state-of-the-art frameworks. Instead, plans are only calculated for the ensemble, defining actions for the collective as such while leaving the execution of actions to be solved by SA–SO.

None of the scheduling, allocation, and execution approaches exploits the flexibility that arises from the possibility of reconfiguration, i.e., the reallocation of resources (capabilities) among the participating agents. In our approach, we already improved the current state of affairs by harnessing this neglected potential, which aids in finding much more robust solutions for scheduling and allocation problems [47]. This also offers the possibility to further improve already-found valid allocations by continuously optimizing the ensemble composition. In contrast to current approaches, we stop the task decomposition on the ensemble level and alternatively let those tasks be solved in an SA–SO fashion by the ensemble of modular reconfigurable robots. This constitutes a new form of the MRTA, integrated with the MARA.

### 6.3. Knowledge Representation and Semantic Hardware Description

In order to access hardware resources and realize complex processes, a connection between abstract capabilities’ description on the level of agents and their concrete execution on actual hardware components was created in our approach. We achieved this by adopting techniques from the field of the semantic web [76], based on RDF [140] and OWL [141] that are suitable for the semantic annotation of hardware components.These technologies are already being used in various projects for the description and execution of capabilities assigned to (hardware) modules [52,142]. In the approach of Reference [52], a capability is assigned to exactly one hardware module and vice-versa. These hardware modules are mounted on UAVs. The UAV itself does not possess explicitly modeled capabilities but rather serves merely as a platform for carrying sensors and actuators. Relationships between the platform and capabilities are expressed in simple terms, such as *canCarry*, in order to outline possible assemblies of components. In the approach of *Dibley* [142], sensors are represented as sensor node agents. The goal of these sensor node agents is to deliver resource provision in terms of monitoring data to other agents that request it. The abstraction of capabilities detached from concrete resources how we integrate it in our approach is not in the focus of the approach of *Dibley* [142]. Moreover, our goal is to span capabilities over different modules to gain new functionality, e.g., a gas sensor and a quadrocopter can have the capability gradient flight, which means to fly along a gradient of measured values, which is not pursued by any of the mentioned other approaches. In our approach, even more complex systems (e.g., quadrocopters) with many sensors and/or actuators have a variety of different capabilities, including mechanisms for their utilization and combination to virtual capabilities. Such more complex descriptions, e.g., the influence of the geometrical position of a sensor and the resulting effects on capabilities, are not taken into account by References [52] or [142] but in the project *KnowRob* [143]. In *KnowRob*, it is not only the geometrical positions of sensors that are regarded as capabilities, but also the relative positions of unknown objects (e.g., a coffee pot) that are determined through sensors. Depending on the categorization of the object, certain actions are performed (e.g., grasping the pot). The information for which actions an object may be utilized is partly derived from the project *Cyc* [144] and the OMICS [144] database. OMICS contains detailed information about possible uses of objects and is expressed in the descriptive language SRDL [143], a semantic extension of URDF [143]. The semantic description in Knowro is directly dependent on the execution. Likewise, relations between capabilities directly mapped to hardware modules are regarded as exclusively additive (e.g., quadrocopter equipped with a camera results in a monitor capability). In addition to those relations, also subtractive dependencies between hardware modules addressed in our approach, e.g., too many (sensor) hardware modules with a high total mass on a quadrocopter may eliminate the capability flying.

The advantages of semantically annotating sensors and actuators with the aforementioned techniques are also included in the definition of the asset administration shell (AAS) from the Industry 4.0 initiative [72]. There, the semantic description of physical objects (e.g., datasheet, 3D model) are made available to the overall system through an interface of the AAS. The AAS enriches each object with semantic annotations, which may be defined using various standards, such as AutomationML [145]. The use of techniques originating in the semantic web [146] is suggested for the semantic interconnection between multiple such standards.

In our approach, we further specified these concepts to employ them for ScORe missions. Thus, common data access protocols such as OPC-UA [147] are employed and extended with a semantic adapter. Subsequently, they are deployed on a commercially available single-board computer or microcontroller that can be attached to real sensors and actuators. On the one hand, this semantic adapter offers an abstract representation of the capabilities that sensors and actuators provide. On the other hand, the semantic adapter combines the descriptive information with its executable capabilities. Compared to all other projects, we enabled the interdependency of resources, i.e., capabilities, in our approach. Focusing on this, capabilities are not bound to single hardware components but emerge from combinations thereof (e.g., gradient flight in Figure 9). The types of dependencies resulting from those combinations are evident, especially when regarding a quadrocopter where subtractive dependencies, as they are mentioned above, have to be taken into account. The goal of our approach concerning modular hardware is to provide a reference system architecture for the definition of capabilities that also describes dependencies between capabilities and can be used with real sensors and actuators. This architecture serves as the basis for the reconfiguration of real hardware in a plug and play manner, which describes itself by means of capabilities.

### 6.4. Related Case Studies

Many projects investigate mobile agents and how they can support emergency management (e.g., Search and Rescue (SAR) scenarios) or environmental research, which both can be classified as ScORe missions. In the following, we discuss related approaches and point out their potential as well as their drawbacks in the context of applying them for handling ScORe missions. Current approaches can roughly be classified into two groups: (1) Approaches that try to map principles found in nature to the development of systems consisting of a multitude of simple robots (called swarm robotic systems [132]), and (2) approaches that use traditional AI techniques (e.g., planning, reasoning, learning, scheduling, etc.) [14,148,149]. Approaches of group (1) are characterized by the fact that agents only have very limited capabilities and are highly specialized for certain tasks, which limits their applicability for ScORe missions, as different ScORe tasks typically require agents with heterogeneous capabilities.

Projects following (1) can mainly be found in the domain of environmental/climate research. Here, the usage of swarm robotic algorithms is more common than in SAR scenarios, as these systems are often only used to collect data but not to make complex online decisions based on it. For instance, in CoCoRo (2011–2014) [39] as well as in its subsequent project subCULTrob (since 2015) [19], autonomous swarms of unmanned underwater vehicles (UUVs) and unmanned surface vehicles (USVs) (all specialized in their capabilities) use swarm robotic principles for ecological monitoring, search, maintenance, exploration, and harvesting in an underwater environment. Other projects focus on developing mechanisms for solving single specialized tasks, e.g., distributed environmental monitoring using mobile USVs. In this domain, the NAMOS project (2003–2007) [150] uses coordination done by a multiagent system, whereas the project CORATAM (since 2014) [18] relies on controllers synthesized with evolutionary algorithms. All approaches classified in type (1) have in common that they reach their goals using algorithms relying on very simple rules (e.g., finding consensus [151], cooperative transport [33,152], or clustering [18]), which can only be achieved by the collective as a whole but not by single agents (often described as the emergent effect [24]). On the one hand, this emergence brings robustness and fault tolerance, as misbehavior of single agents can easily be compensated for within a homogeneous swarm where all agents are identical. On the other hand, in complex applications involving multiple tasks (e.g., ScORe missions), problems occur after achieving the goal the swarm robotic system is trained for, as these systems rely on additional guidance if interdependent decisions or actions must be made [49]. In addition, agents used in subsequent tasks usually need to have different capabilities. This assumption is often not considered in these approaches, as the swarms usually consist of homogeneous agents.

Approaches of group (2) appear to be more appropriate for complex scenarios. The Swarmanoid project (2006–2010) [23] combines multiple mechanisms from (1), e.g., to solve a precisely predefined SAR mission using heterogeneous agents, but therefore uses explicit scripted behavior. Other projects for handling SAR scenarios often combine intelligent autonomous agents with different capabilities for supporting humans in the SAR process. For example, the SHERPA project (2013–2017) [48] focuses on human–robot interaction with UAV and unmanned ground vehicles (UGVs). The SWARMIX project (2011–2014) [36] combines human capabilities with those of animals (e.g., dogs), as well as artificial agents (e.g., UAV) to create heterogeneous SAR systems. Systems designed for SAR scenarios consisting of artificial agents only are often restricted in their autonomy, as they are only able to react to predefined events. In SINUS (2013–2015) [14] or AirShield (2008–2011) [12], a wireless connection can be autonomously established over a long distance to stream data from an area of interest to a distant operator for further evaluation. This evaluation and an appropriate reaction have to be determined by the human user of the system.

All of the described approaches have in common that they are heavily specialized for their dedicated application and lack the ability to autonomously adapt to changing requirements (i.e., new capabilities are demanded from agents), which is very important in ScORe missions. While certain approaches use SA and SO to adapt to a changing environment, the possibility of reallocating the agents’ capabilities is neglected. This hinders ongoing approaches to actually adapt to changing ScORe tasks in ScORe missions or switch from one ScORe mission to another (e.g., from environmental monitoring to SAR). Our approach instead allows for both kinds of adaptation. By combining SA and SO principles with planning, we suggest a new paradigm of instructing an ensemble. Being able to reallocate capabilities seems to be the next step to enable real-world applications. We, therefore, will make this additional degree of freedom utilizable and consequently will consider it throughout all software layers. The resulting system architecture will allow robot ensembles to deal with ScORe missions in general.

### 6.5. Conclusion on the State-of-the-Art

As shown above, many relevant aspects have already been investigated to a certain level. However, the aspect of reconfigurable agent capabilities has not gained appropriate attention until now. Exploiting this possibility becomes a key enabler for employing a new paradigm for planning and executing tasks in the context of ScORe missions. With this approach, the problem of handling uncertainties in real-world environments gets compensated by the self-organization abilities of the ensemble. In our approach, we researched the required basic principles concerning the impact of self-organized reconfiguration on the capability level as well as the necessary self-descriptive hardware components. Additionally, this overcomes the issue of specialization current approaches suffer from and enables self-adaptation and self-organization of the system when switching ScORe tasks or even ScORe missions by enabling multipotent systems.

## 7. Conclusions

We presented a layered reference software architecture that enables multipotent systems and merges the benefits of both homogeneous and heterogeneous systems while overcoming their drawbacks. Thus, we showed how we integrate all necessary algorithms in that architecture. In the scope of this paper, we put the main focus on depicting the interplay of those techniques and algorithms. Robots implementing the proposed software can become heterogeneous specialists during runtime while they are entirely homogeneous at design time. We further provided proof of concepts and summarized preliminary results we achieved with our approach so far. Therefore, we included demonstrations of how our reference architecture can be used to control and coordinate robots under real-world conditions. We further illustrated the feasibility of physical reconfigurations that is the core concept of our approach. Our experiments showed that reconfigurations can be calculated autonomously by the ensemble itself either in a centralized or in a distributed fashion. We showed that these reconfigurations can even be calculated with higher numbers of hardware modules but need further problem decomposition when the number of agents in an ensemble gets bigger.

Our current and next steps include the design and implementation of (1) a domain-specific language for specifying modular, hierarchical task networks (HTN) in the domain of ScORe missions containing primitive tasks on collective level combined with their corresponding ensemble tasks for their execution. Additionally, we plan to (2) design and implement an adaptive, distributed, and transactional algorithm to transfer task execution knowledge in the case of failure to increase the robustness of the system. Further, we aim at introducing (3) a mechanism to achieve consensus within the ensemble, e.g., during synchronization of different planning results on the task layer in Section 4.1, we will appropriately adapt a leader election approach [62] meeting all needed requirements defined in our system class. Further, we will (4) automate the act of planning by enabling our system to learn HTNs [153] from data provided by experts, so that learned valid plans are suggested to the system’s user. Additionally, we plan to (5) extend existing ontologies to be used within our semantic hardware layer for a smooth integration and usage of hardware capabilities, (6) develop a middleware for the management of all available SDDs (even if they are not connected) to “suggest reconfigurations proposals” (cf. Figure 5), and (7) enhance the prototypes to be used with the sensors and actuators of our use case, described in Section 2. We will also put further focus on the exchange of actuators encapsulated in an SDD to even convert a robot from being able to fly (enabled by an SDD encapsulating, e.g., a quadrocopter) to one being able to drive or swim when this is necessary during a ScORe mission.

## Abbreviations

The following abbreviations are used in this manuscript:

AAS	Asset administration shell
AGL	Above ground level
AI	Artificial intelligence
BASF	Badische Anilin und Soda Fabrik
BDI	Belief desire intention
CSOP	Constraint satisfaction (and optimization) problem
DTS	Distributed temperature sensing
HTN	Hierarchical task network
LTE	Long term evolution
MARA	Multiagent resource allocation problem
MAS	Multiagent system
MRTA	Multirobot task allocation problem
NBL	Nocturnal boundary layer
NP	Nondeterministic polynomial time
OPC-UA	Open platform communication-unified architecture
PDDL	Planning domain definition language
RAP	Resource allocation problem
ROS	Robot operating system
SA	Self-adaptation
SAR	Search an rescue
ScORe	Search (S), continuously Observe (cO), and React (Re)
SDD	Self-descriptive device
SO	Self-organization
TRANSFORMRS	Task and resource allocation strategy for multi robot systems
UAV	Unmanned aerial vehicle
